# Repression of oxidative phosphorylation by NR2F2, MTERF3 and GDF15 in human skin under high-glucose stress

**DOI:** 10.1016/j.redox.2025.103613

**Published:** 2025-03-27

**Authors:** S. Ley-Ngardigal, S. Claverol, L. Sobilo, M. Moreau, C. Hubert, J. Goupil, A. Poulignon, W. Mahfouf, H. Fatrouni, L. Dard, M. Juan, L. Gales, A. Merched, C. Tokarski, E. Leblanc, A. Galinier, D. Lacombe, H.R. Rezvani, F. Bellvert, K. Pays, C. Nizard, N. Dias Amoedo, A.L. Bulteau, R. Rossignol

**Affiliations:** a INSERM U1211, 33076, Bordeaux, France; b Bordeaux University, 146 rue Léo Saignat, 33076, Bordeaux, France; c LVMH Recherche, Saint-Jean-de-Braye, France; d Univ. Bordeaux, Bordeaux Proteome, Bordeaux, France; e CELLOMET, ADERA, 146 rue Léo Saignat, 33076, Bordeaux, France; f INSERM UMR 1312, Bordeaux Institute of Oncology (BRIC), Bordeaux, France; g Metabolomics facility METATOUL, Toulouse, France; h Univ. Bordeaux, CNRS, Bordeaux INP, CBMN, UMR 5248, Pessac, F-33600, France; i RESTORE, UMR 1301-Inserm 5070-CNRS EFS Univ. P. Sabatier, Toulouse, France; j Medical Genetics Department, CHU Bordeaux, 33076, Bordeaux, France

**Keywords:** Hyperglycemia, Oxidative phosphorylation, Skin, MTERF3, GDF15

## Abstract

Lifestyle factors such as a Western diet or metabolic diseases like diabetes disrupt glucose homeostasis and induce stress responses, yet their impact on skin metabolism and structural integrity remains poorly understood. Here, we performed multiomic and bioenergetic analyses of human dermal fibroblasts (HDFs), human equivalent dermis (HED), human reconstructed skin (HRS), and skin explants from diabetic patients. We found that 12 mM glucose stress represses oxidative phosphorylation (OXPHOS) through a dual mechanism: the glucose-dependent nuclear receptor NR2F2 activates mitochondrial transcription termination factor 3 (MTERF3) while inhibiting growth-differentiation factor 15 (GDF15). Promoter assays revealed that MTERF3 is regulated by NR2F2 and MYCN, whereas GDF15 is modulated by NR2F2 and FOS. Consequently, OXPHOS proteins and mitochondrial respiration were suppressed, and MTERF3 overexpression additionally interfered with collagen biosynthesis. In contrast, GDF15 supplementation fully rescued hyperglycemia-induced bioenergetic and metabolomic alterations, suggesting a pharmacological strategy to mitigate hyperglycemic damage in the skin. Finally, silencing GDF15 or TFAM impaired fibroblast haptotaxis and skin reconstruction, underscoring the crucial role of mitochondrial energetics in dermal structure and function. Collectively, these findings identify the NR2F2–MTERF3–GDF15 axis as a key mediator of OXPHOS suppression and highlight a potential therapeutic target to preserve skin integrity under hyperglycemic stress.

## Highlights

• 12 mM glucose stress represses oxidative phosphorylation in human dermis and reconstructed skin.
• NR2F2 activates MTERF3 and inhibits GDF15 upon hyperglycemia.
• NR2F2, FOS, MYCN, ATF3 and ATF4 combine to repress OXPHOS in human skin.
• Nanomolar GDF15 supplementation activates mitochondrial respiration in the dermis.
• GDF15 silencing abolishes human skin reconstruction and alter mitochondrial network.

## Graphical abstract

**Figure:**
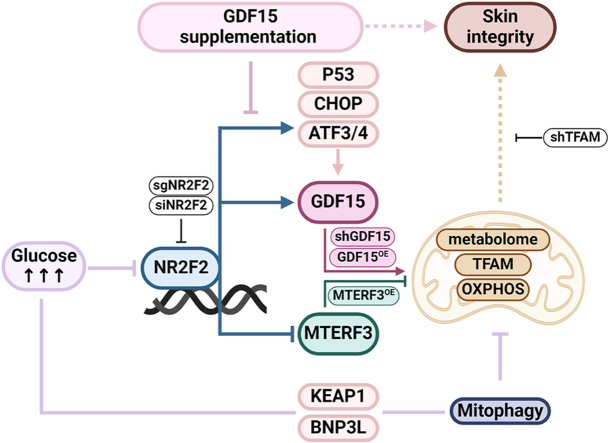
We identify the NR2F2-GDF15-MTERF3 axis as a key regulatory mechanism that represses oxidative phosphorylation in human skin under high-glucose stress. This repression profoundly impacts collagen organization and overall skin physiology. By integrating both bioenergetic and structural responses, the NR2F2-GDF15-MTERF3 axis supports skin resilience, enabling tissues to adapt to hyperglycemia. These findings reveal new insights into how skin homeostasis is maintained under metabolic stress and highlight potential therapeutic targets for improving skin integrity in hyperglycemic conditions.

## 1 Introduction

Glucose is vital for mammalian cells, fueling energy production, storage, and the biosynthesis of nucleotides, amino acids, fatty acids, and glutathione. Its sensing, transport, and metabolism are regulated by interconnected pathways (e.g., Pi3K/Akt, insulin, Hif1α, cAMP/PKA, ChREBP, mTOR, RAS, and AMPK) in a tissue-specific manner. While liver, brain, heart, and muscle glucose metabolism is well characterized, the skin remains understudied despite its unique functions in collagen production, temperature regulation, and hair growth. Dermal fibroblasts are central to skin structure, generating connective tissue and supporting nutrient diffusion. According to the World Health Organization, normal fasting blood glucose ranges from 3.9 to 5.6 mM, and levels above 6.9–12 mM indicate hyperglycemia. Because dermal glucose concentrations mirror blood levels [1], skin fibroblasts can experience glucose overload in high-glucose diets, metabolic diseases, or during “stress hyperglycemia” in hospitalized patients [2], even without diabetes. However, how the dermis adapts to such stress and whether high glucose induces molecular changes in skin metabolic machinery remains unclear. Studies on hyperglycemia-related bioenergetics have shown a “glycolytic shift” [[3], [4], [5]], yet the precise mechanisms linking elevated glucose to mitochondrial repression—beyond changes in mitochondrial network morphology [6]—are still unclear. Biochemical research has identified negative regulators of mitochondrial biogenesis, including mitochondrial transcription termination factor 3 (MTERF3) and nuclear receptor corepressor 1 (NCOR1), but their roles in high-glucose stress are not well understood [7,8].

Here, we examined metabolic reprogramming in human skin fibroblasts exposed to 12 mM glucose, both in vitro and in reconstructed skin. We discovered that the NR2F2–MTERF3–GDF15 axis is involved in hyperglycemia-induced repression of oxidative metabolism in the skin, suggesting potential roles for MTERF3 and GDF15 in glucose homeostasis and dermal resilience.

## 2 Material and methods

### 2.1 Ethical compliance

For cell culture studies, skin samples were collected from anonymous adult patients who have undergone plastic surgeries performed by independent surgeons. Surgical residues were harvested according to French regulations (agreement DC-2023-5824) and after obtaining written informed consent from each donor.

### 2.2 Cell and tissue culture

*Cell culture maintenance and treatment.* Primary dermal fibroblasts were obtained from three donors: a 25 years old female (ThermoFisher #C-013-5C, Lot #2186199) a 46 years old female (ATCC CRL-2106; FHN6609) and a 23 years old female (ATCC CRL-1947; FHN4927). The human skin fibroblasts (HSF) were taken at abdominal location. They were then grown in Dulbecco's Modified Eagle Media (DMEM, Gibco) containing 5.55 mM glucose (1 g/L) and supplemented with 10 % fetal bovine serum (Gibco), 100 U/ml penicillin and 100 U/ml streptomycin (Gibco), in 5 % CO_2_ at 37 °C. For all experiments, the cells were incubated with different sugar concentration and the study was performed during the exponential phase of growth, at 70 % confluency. Cell cultures maintained in 5.55 mM glucose served as the control condition, reflecting normal fasting blood glucose levels (3.9–5.6 mM). A medium containing 6.5 mM glucose was designated as “mild hyperglycemia,” whereas 12 mM and 25 mM glucose media were considered “strong hyperglycemia” and “supraphysiological hyperglycemia,” respectively. The human primary skin fibroblasts cell lines were generated on the same day and the cells underwent similar number of passages at the moment of the various experiments. The treatment consisted of 48h of incubation in DMEM containing 5.55 mM, 6.5 mM, 12 mM or 25 mM of glucose or 5.55 mM of galactose. In some condition the medium was supplemented with purified human GDF15 (#Qk017, QKine). Primary human epidermal keratinocytes were purchased from tissue and cell bank of the Lyon Hospital Center. The cells were then grown in EpiLife (Gibco) supplemented with human keratinocyte growth factor (HKGS), in 5 % CO_2_ at 37 °C.

*Cell transfections.* Cell transfection was carried out with Lipofectamine 3000 according to the manufacturer's recommendations (ThermoFisherFisher, #L3000001). In 96-wells plate, 1.10^4^ human primary dermal fibroblasts were seeded and the following day the cells were transfected with 500 ng of plasmid DNA or 20 nM of esiRNA. The cells were then studied by biochemistry or imaging after 48h of transfection. The plasmids were purchased from Addgene: pHAGE-mt-mKeima (#131626), pAAV-HyPer3 (#119183) and control empty vector (#91980). The esiRNAs were purchased from Sigma-Aldrich: esiRNA human FOS (#EHU034291), esiRNA human MTERF3 (#EHU056621), esiRNA human MYCN (#EHU093561), esiRNA human NR2F2 (#EHU023641), esiRNA human GDF15 (#EHU052901) and esiRNA control (#SIC001).

*Generation of GDF15*^*silencing*^*, MTERF3*^*overexpression*^*and GDF15*^*overexpression*^*mutant human skin fibroblasts*. Expression plasmids in lentiviral vectors were purchased for MTERF3 Human Tagged ORF Clone (#RC201030L4, Origene) and GDF15 Human Tagged ORF Clone (#RC201295L2, Origene). Lentiviral particles were produced by Vect'UB platform (CNRS 3427 - INSERM US00S). Lentiviral vector was produced by transient transfection of 293T cells according to standard protocols. In brief, subconfluent 293T cells were cotransfected with lentiviral genome (psPAX2) [9], with an envelope coding plasmid (pMD2G-VSVG) and with vector constructs by calcium phosphate precipitation. LVs were harvested 48 h post transfection and concentrated by ultrafiltration. Viral titers of pLV lentivectors were determined by transducing 293T cells with serial dilutions of viral supernatant and EGFP expression was quantified 5 days later by flow cytometry analysis or provirus copies number by qPCR method. GDF15 Human shRNA Lentiviral Particle (#TL312802V, Origene) were directly purchased. 80 000 cells were incubated with viral supernatants (MOI 10) for 48 h at 37 °C. The clones overexpressing *GDF15* or *MTERF3*, and the clones with GDF15 silencing were obtained by sorting the cells using the GFP expression and following puromycin treatment 1 μg/mL during 3 days.

*Generation of Gaussia-luciferase promoter activation reporter assay for MTERF3 and GDF15- in human skin fibroblasts.* GLuc-ON promoter reporter plasmids in lentiviral vectors were purchased for MTERF3 (#HPRM41675-LvPG02, GeneCopoeia) and GDF15 (#HPRM45772-LvPG02, GeneCopoeia). Lentiviral particles were produced by Vect'UB platform (CNRS 3427 - INSERM US00S). Lentiviral vector was produced by transient transfection of 293T cells according to standard protocols. In brief, subconfluent 293T cells were cotransfected with lentiviral genome (psPAX2) [9], with an envelope coding plasmid (pMD2G-VSVG) and with vector constructs by calcium phosphate precipitation. LVs were harvested 48 h post transfection and concentrated by ultrafiltration. Viral titers of pLV lentivectors were determined by transducing 293T cells with serial dilutions of viral supernatant and EGFP expression was quantified 5 days later by flow cytometry analysis or provirus copies number by qPCR method. 80 000 cells were incubated with viral supernatants (MOI 10) for 48 h at 37 °C. The clones expressing *GDF15* or *MTERF3* promoter reporter were obtained by puromycin treatment 1 μg/mL during 3 days. GLuc-ON promoter reporter dermal fibroblasts were seeded in 96-well plate for luminescence (#165306, ThermoFisher) and immediately transfected by esiRNA previously described. These cells are incubated during 24h with 5.55 mM/12 mM/25 mM of glucose or 5.55 mM of galactose. Then, cells are incubated during 15min with 5 μM of coelenterazine h (#C6780, ThermoFisher). Absorbance was measured at 466 nm and cells were fixed for normalization by using the Sulforhodamine B assay [10].

*Generation of NR2F2*^*KO*^*human skin fibroblasts using CrisprCas9.* Two plasmids encoding sgRNA (silencing guide RNA) targeting NR2F2 (NCBI 7026) and one plasmid encoding sgRNA control were designed by the “CRISPR’Edit” of the TMB-Core of the Bordeaux University (CNRS 3427 - INSERM US00S). ZsGreen vector was used. NR2F2-sgRNA1 is CGGGCAGTTCGCGCTGACCA and NR2F2-sgRNA2 is TATATCCGGACAGGTACGAG. Lentiviral particles were produced by Vect'UB platform (CNRS 3427 - INSERM US00S). Lentiviral vector was produced by transient transfection of 293T cells according to standard protocols. In brief, subconfluent 293T cells were cotransfected with lentiviral genome (psPAX2) [9], with an envelope coding plasmid (pMD2G-VSVG) and with vector constructs by calcium phosphate precipitation. LVs were harvested 48 h post transfection and concentrated by ultrafiltration. Viral titers of pLV lentivectors were determined by transducing 293T cells with serial dilutions of viral supernatant and ZsGreen expression was quantified 5 days later by flow cytometry analysis or provirus copies number by qPCR method. Primary dermal fibroblasts were then transduced by the lentiviruses produced. The cells were then sorted, and verification of knockout carried out by quantitative PCR and Western Blot.

*Generation of Human Equivalent Dermis (HED) and Human Reconstituted Skin (HRS).* HRS production lasts 38 days. Firstly, the dermis is regenerated by seeding human dermal primary fibroblasts on a dermal scaffold made of collagen-matrix (Integra LifeSciences, Princeton, NJ, USA). Dermal substrate is placed on inserts designed for 12-well plates (Thincert Griener). This Dermal Equivalent model is cultured for 21 days in DMEM (Gibco) containing 5.55 mM or 12 mM glucose and supplemented with 10 % fetal bovine serum (Gibco), 100 U/ml penicillin and 100 μg/mL streptomycin (Gibco), 1 μg/mL Amphotericin B (Sigma-Aldrich) in 5 % CO2 at 37 °C. This medium is supplemented with 50 μg/mL Vitamin C (Sigma-Aldrich) and is changed every day.

Secondarily, at d21, epidermal primary keratinocytes are seeded on HED. Immerged HRS is grown for 3 days in DMEM (Gibco) – Ham F12 (Gibco) in ratio 2:1 supplemented with 5.55 mM or 12 mM glucose, 10 % fetal bovine serum (Gibco), 100 U/ml penicillin and 100 μg/mL streptomycin (Gibco), 1 μg/mL Amphotericin B (Sigma-Aldrich), 0.4 μg/mL Hydrocortisone (Sigma-Aldrich), 24.3 μg/mL Adenine (Sigma-Aldrich), 0.4 μg/mL Isoproterenol (Sigma-Aldrich), 10 μg/mL Insulin (Sigma-Aldrich), 2 nM Triiodo-l-thyronine (Sigma-Aldrich) and 10 ng/mL Epidermal Growth Factor (Sigma-Aldrich) in 5 % CO2 at 37 °C. The medium of immerged HRS is changed every day. Finally, early reconstituted skin is elevated into air-liquid interface in new deep-well plate (Thincert Griener) at d24. Only the dermis is in contact with Phenion® Air-Liquid Interface medium (Phenion, Henkel) completed with 5.55 mM or 12 mM of glucose and the medium is changed 3 times by week. At d38, mature HRS are harvested and one-half of every model is analyzed by biochemistry, immunohistochemistry, and immunofluorescence. Each condition was produced in triplicate for all analysis. At J38, final day, HRS were analyzed by biochemistry, immunohistochemistry, and immunofluorescence.

*Cell Proliferation Assays for Live-Cell Analysis.* Human Dermal Fibroblasts were seeded in an IncuCyte® ImageLock 96-well plates at 5000 cells per well. The medium containing the different glucose concentrations were added into the corresponding wells. The proliferation assay was monitored by the IncuCyte S3 live-cell analysis system (Essen BioScience, Ltd., Royston Hertfordshire, UK) up to 7 days. The images were obtained every 6 h. Cell proliferation was determined as percent confluence from phase images and was analyzed by IncuCyte image analysis software (Sartorius).

*Haplotaxis Migration Assays.* Human Dermal Fibroblasts were seeded in migration chambers (#662638, GREINER BIO-ONE) in 24-well plates at 50 000 cells per well. These cells were grown in serum-free medium in the upper chamber and transfection with esiRNA was performed as described below. Culture medium with serum and different glucose concentration (5.55 mM, 12 mM or 25 mM) was added in the lower chamber. Cells were incubated during 48 h and the medium was removed. Cells were washed with PBS, then fixed with 4 % paraformaldehyde during 2 min. Cells were washed with PBS and permeabilize by 100 % methanol during 10min. Cells were washed with PBS and stained with crystal violet during 15 min. Cells were washed with PBS and the non-migrated cells remaining on the upper chamber were scrapped with cotton swabs. The migrated cells were imaged under a light microscope. The cell migration was determined as percent confluency using ImageJ image analysis software.

### 2.3 Molecular biology

*RNA extraction, cDNA synthesis and real time quantitative PCR (qPCR).* RNAs were extracted from dermal fibroblasts using Monarch Total RNA Miniprep Kit (#T2010S, New England Biolabs) following the manufacturer's recommendations. Total RNA was quantified spectrophotometrically, and RNA quality was verified using Agilent RNA 6000 Nano kit on an Agilent 2100 Bioanalyzer (Agilent Technologies). cDNA synthesis was performed using SuperScript VILO Master Mix (#11755050, ThermoFisher). Gene expression analysis was performed using 7500 Real Time PCR System (Applied Biosystems) and Master Mix TaqMan Fast Advanced (#4444963, ThermoFisher). TaqMan Gene Expression Assay Probes were purchased from Applied Biosystems (#4448892, ThermoFisher). We used probes marked with FAM for our specific targets: TFAM (Hs01082775_m1); MTERF3 (Hs00210971_m1); GDF15 (Hs03986124_s1); NR2F2 (Hs00819630_m1); COQ9 (Hs00941291_m1); FOS (Hs04194186_s1); MT-RNR1 (Hs02596859_g1); PGC1α (Hs00173304_m1); ATF4 (Hs00909569_g1); ATF3 (Hs00231069_m1); CHOP (Hs99999172_m1); P53 (Hs01034249_m1); MAPK1 (Hs01046830_m1); MAPK3 (Hs00385075_m1); MMP2 (Hs01548727_m1); FN1 (Hs01549976_m1); P16 (Hs00923894_m1) and P21 (Hs00355782_m1). We used endogenous probes marked with VIC for endogenous control: HPRT1 (Hs03929096_g1); β2M (Hs06637353_s1) and GUSB (Hs04421153_s1). The comparative 2^-ΔΔCt^ method was used to compare changes in gene expression levels. First, the ΔCt was calculated (Ct_TARGET_ - Ct_GUSB_) then the ΔΔCt (ΔCt_TREATED 12mM_ - ΔCt_TREATED 5.55m_). Results were expressed as a relative quantification (2^-ΔΔCt^), indicated as Arbitrary Units (A.U.).

*Taqman Low Density Array (TLDA) real-time RT-PCR.* The HRS were crushed with the Precellys Evolution and its refrigerated chamber (Cryolys). The protocol used was 3 × 23s/6300 rpm/2 min break. The lysis buffer consistsed of the RA1 solution (#740955.50, Macherey-Nagel) and 1 % β-mercaptoethanol. Separation of proteins/nucleic acids was obtained with a solution of Trizol (r) (1 mL for 100 mg of tissue) for 5 min then chloroform for 3 min. After centrifugation for 15 min at 12 000×*g* at 4 °C, the aqueous phase containing the total RNAs was recovered. Then the RNAs were purified according to the kit recommendations (#740955.50, Macherey-Nagel). Reverse transcription was performed with High Capacity Reverse Transcription Kit (#4368813, Life Technologies-Applied). The mix TLDA used was Taqman Gene expression Master Mix (#4369016, Life Technologies-Applied). TLDA plate used were 48 genes card (Life Technologies-Applied). Real-time qPCR was achieved with the 7900HT Fast Real-Time PCR System with 96 Well Block Module (Applied Biosystems). Data were normalized to the beta-2-microglobuline gene expression. Results were expressed as a relative quantification (2-ΔΔCt), indicated as Arbitrary Units (A.U.).

*Assay for Transposase-Accessible Chromatin with high throughput sequencing (ATAQ seq).* Overall, 5.10^4^ dermal fibroblasts were seeded in 12-well plates and incubated during 48h in 5.55 mM glucose, 12 mM glucose or 5.55 mM glucose +100 nM purified GDF15 with 3 replicates per conditions. Library were prepared by following Diagenode kit instructions (#C01080001, Diagenode): cell lysis, nuclei extraction, tagmentation of the 9 libraries (each with a different tag) and DNA purification (#C01011032, Diagenode), library amplification, size selection and library quality control. The 9 libraries were pooled. Library was sequenced by high-throughput sequencing on NovaSeq™6000 S2 in “Paired-End” reading of 2 times 100 nucleotides by IntegraGen (Evry). The datas were analyzed with Galaxy, an open-source (eweb-based platform (usegalaxy.org). We used the Galaxy Europe server for the analysis of ATAC-Seq data following the “ATAC-Seq data analysis” analysis pipeline (https://training.galaxyproject.org/training-material/topics/epigenetics/tutorials/atac-seq/tutorial.html#atac-seq-data-analysis). Mapping was performed using Cutadapt (Galaxy version 1.16.5). The clean sequences were aligned against the Canonical hg38 human genome via the Bowtie2 module (Galaxy version 2.4.2). Low mapping quality reads were filtered using (Galaxy version 2.4.1). All datas were normalized per quantile with R package preprocessCore (normalize.quantiles). Filtering of Mapped Reads was performed using MarkDuplicates (Galaxy version 2.18.2.2) andcPeak calling. Was performed using MACS2 (Galaxy version 2.1.1.20160309.6) and BED converter (Galaxy version 2.30.0). Coverage Visualisation was performed using deepTools, plotHeatmap, computeMatrix, pyGenomeTracks (Galaxy version 3.6) and Circos (Galaxy Version 0.69.8).

### 2.4 Biochemistry

*Protein extraction for proteomic analysis and label-free quantitative proteomics.* Human dermal fibroblasts cell pellets were lysed using a lysis buffer containing RIPA 1X buffer (#R0278, Sigma-Aldrich) supplemented with 1:100 Phosphatase inhibitor (#P0044, Sigma-Aldrich) and 1:100 Protease inhibitor cocktail (#P8340, Sigma-Aldrich). Protein lysates were placed on ice for 15 min, vortexed and cleared by centrifugation at 12 000×*g* for 15 min at 4 °C. Protein concentration was evaluated using the BCA Protein Assay kit – Reducing Agent Compatible according to the manufacturer's instructions (#23250, ThermoFisher). Protein extracted were dissolved in Laemli buffer (#1610747, Bio-Rad) containing 4 % β-mercaptoethanol (#M3148, Sigma-Aldrich) by incubation for 5 min at 95 °C. Protein solution at 2 μg/μL was prepared. This analysis was performed by the proteomics core facility at University of Bordeaux (https://proteome.u-bordeaux.fr/en/). The steps of sample preparation, protein digestion and nano-liquid chromatography–tandem mass spectrometry analysis were performed as previously described [5]. For protein identification, Sequest HT algorithm through Proteome Discoverer 2.5 Software (ThermoFisher Scientific Inc.) was used to search against a *Homo sapiens* database (78 806 entries, Reference Proteome Set, release 2021_01). Database was downloaded from http://www.uniprot.org/website. Two missed enzyme cleavages were allowed. Mass tolerances in MS and MS/MS were set to 10 ppm and 0.02 Da. Oxidation of methionines, methionine loss, methionine loss with acetylation and protein N-terminal acetylation were searched as dynamic modifications. Carbamidomethylation on cysteine was searched as static modification. Peptide validation was performed using Percolator algorithm [14] and only “high confidence” peptides were retained corresponding to a 1 % False Positive Rate at peptide level. Peaks were detected and integrated using the Minora algorithm embedded in Proteome Discoverer. Normalization was performed based on total human peptide amount. Protein ratio were calculated as the median of all possible pairwise peptide ratios. A *t*-test was calculated based on background population of peptides or proteins. A *t*-test p-value less than 0.05, a minimum of two peptides matched to a protein, and a ≥2- fold change in relative abundance between the two conditions (n = 3 in each group) were used as the criteria for identification as a differentially expressed protein. Noticeably, only unique peptides were considered for calculation at protein level. Analysis of the data was performed using Ingenuity Pathway Analysis (Qiagen). For the comparison of MTERF3 and GDF15 impact on the proteome, Heatmaps were established using the R package "pheatmap", R Studio version 2024.04.0 + 735. 2280 proteins were associated with rows and clustered using the K means method (k = 50). All values correspond to a relative abundance ratio under different conditions for each studied protein. Hierarchical clustering was performed using the ‘pheatmap’ R statistical package, with a Correlation distance matrix and "ward.D2" clustering method. Quantile breaks were obtained using the “ggplot2” package.

*Automated capillary electrophoresis western analysis*. Human dermal fibroblasts (HDF), human equivalent dermis (HED) or human reconstructed skin (HRS) were lysed in whole cell extraction buffer (25 mM Tris HCL buffer pH 7.5; 0.25 M saccharose; 0.2 mM MgSO4; 20 mM EDTA; 0.4 % Triton X-100; 2 mM DTT) supplemented with protease inhibitor cocktail (Complete Mini; Roche, Boulogne-Billancourt, France) and phosphatase inhibitor cocktail (PhosSTOP, Sigma-Aldrich). Fibroblasts lysates were sonicated for 5 cycles of 8 s ON/30 s OFF, HED ad HRS lysates were sonicated for 10 cycles of 8 s ON/30 s OFF with a Bioruptor Pico Ultrasonicator (Diagenode, France) at 4 °C, then spun down for supernatant harvest. The supernatants were retrieved and frozen at −80 °C until use**.** Protein concentration was determined by the DC protein assay (Bio-Rad laboratories, Hercules, CA).

Protein samples were separated by capillary electrophoresis using the 12–230 kDa Wes Separation Module capillary cartridges in the Simple Protein Wes system (SM-W004, ProteinSimple) following the manufacturer's protocol. Adequate protein concentrations and antibody dilutions were determined in preliminary assays in order to allow optimal quantitative conditions. The following primary antibodies were used: complex I NDUFB8 (#NBP1-88859, Novus) 1/100; complex II SDHB (#NBP1-87069, Novus) 1/20; complex III UQCRC2 (#NBP1-80861, Novus) 1/50; complex IV COX4 (#NB110–39115SS, Novus) 1/25; complex V ATP5A (#NBP2-67170, Novus) 1/50; GDF15 (#ab206414, Abcam) 1/20 and MTERF3 (#ab230232, Abcam) 1/250. The area of specific peaks of target proteins was determined using Compass software (Protein Simple) and normalized against the total protein detected with the total protein detection module kit ((DM-TP01, ProteinSimple). Secondly, the data were expressed as a percentage with 100 % for the condition without treatment (5.55 mM glc).

*Quantification of Coenzyme Q10 content (reduced and oxidized forms).* The specific determination of absolute CoQ10 content and its steady-state redox status by HPLC-EC measurement was adapted from [11]. Isotonic homogenates of one million of cultured cells were extracted in isopropanol, after intense agitation and centrifugation, the supernatrant was directly injected in the analytical system which consists of an isocratic pump and an electrochemical detector ECD-3000 RS from ThermoScientific. The two separated reduced and oxidized forms at the outlet of the C18 column were subject to a successive reduction then oxidation potentials generating the detected electron flow proportional to the quantity of molecules. The calibration was carried out from oxidized coenzyme Q10 purchased from SIGMA and the reduced form obtained by electrical reduction of the latter. According to this procedure the detection limit is of the order of 1 nM.

*Metabolic profiling by IC-HRMS & NMR 1D*^*1*^*H*. Fibroblast cells were grown in 6-well plates in DMEM containing 5 mM or 12 mM glucose, 12 mM glucose and 100 nM purified GDF15. Cells used were skin fibroblasts wildtype, or genetically modified as follow: GDF15^silencing^, MTERF3^overexpression^ and GDF15^overexpression^. Cells were rinsed 3 times with PBS, before being sealed and quenched in liquid nitrogen. They were extracted using cold extraction solution. A scraping step was included to help detaching the cells from the wells. After incubation, the tubes containing the extracts were evaporated and the cell pellets stored at −80 °C. Addition of 50 μL of IDMS (Isotope Dilution Mass Spectrometry) then vortexed. Samples were re-suspended in 100 μL MilliQ water, centrifuged and the supernatant were transferred in a MS vial for mass spectrometry analyses. In order to control the quality of the analysis, Blank samples are done for each type of sample.

*Analysis of cellular central metabolites by high resolution mass spectrometry (HRMS).* The analyses were carried out on an IC-MS platform of a liquid anion exchange chromatography Dionex™ ICS-5000+ Reagent-Free™ HPIC™ (ThermoFisher Scientific™, Sunnyvale, CA, USA) system, coupled to a to an Orbitrap Qexactive + mass spectrometer (ThermoFisher Scientific, Waltham, MA, USA) equipped with a heated electrospray ionization probe. Liquid anion exchange chromatography was performed with the ThermoFisher Scientific Dionex ICS-5000+ Reagent-Free HPIC system (ThermoFisher Scientific) equipped with an eluent generator system (ICS-5000+EG, Dionex) for automatic base generation (KOH). Analytes were separated within 50 min, using a linear KOH gradient elution applied to an IonPac AS11-HC column (250 × 2 mm, Dionex) equipped with an AG11-HC guard column (50 × 2 mm, Dionex) at a flow rate of 0.38 mL/min. The gradient program was following: equilibration with 7 mM KOH during 1.0 min; then KOH ramp from 7 to 15 mM, 1–9.5 min; constant concentration 10.5 min; ramp to 45 mM in 10 min; ramp to 70 mM in 3 min; ramp to 100 mM in 0.1 min; constant concentration 8.9 min; drop to 7 mM in 0.5 min; and equilibration at 7 mM KOH for 7.5 min. The column and autosampler temperatures were ThermoFisherstated at 25 °C and 4 °C, respectively. The injected sample volume was 15 μl. Measures were performed in triplicates from separate cultures.

High-resolution experiments were performed with an ICS5000+, ion chromatography system (Dionex, CA, USA) system coupled to an Orbitrap Qexactive + mass spectrometer (ThermoFisher Scientific, Waltham, MA, USA) equipped with a heated electrospray ionization probe. MS analyses were performed in negative FTMS mode at a resolution of 140 000 (at 400 *m*/*z*) in full-scan mode, with the following source parameters: the capillary temperature was 325 °C, the source heater temperature, 380 °C, the sheath gas flow rate, 50 a.u. (arbitrary unit), the auxiliary gas flow rate, 5 a.u., the S-Lens RF level, 50 %, and the source voltage, 2.75 kV. Metabolites were determined by extracting the exact mass with a tolerance of 5 ppm. Data processing was performed with TraceFinder 4.1 software.

*Analysis of extracellular metabolites by NMR Analysis.* Samples of 250 μl supernatant were collected. A mix of 180 μL of sample and 20 μl of TSP-d4 (Sodium-2,2,3,3-trimethylsilylpropionate D4) at 9.050 mM in D2O, is done and 175 μL of this mix is transferred into a 3 mm NMR Tubes. The acquisition of 1D 1H NMR spectra was done on an Avance Neo 800 MHz equipped with a CQPCI 5 mm probe (Z-Gradient, ^1^H, ^13^C, ^31^P, ^15^N, ^2^H). Following parameters were used for the acquisition.

**Table:** 

Parameters	Values
Pulse program	zgpr30
Pulse angle	30°
Time Domaine (TD)	65536
Number of dummy scan	4
Number of scan	64
Acquisition time	2.0316160
Pulse P1 length(μsec)	7.98
Pulse P1 power(dB)	−12.14
Pulse P9 power(dB)	43.80
Acquisition temperature	280 K

Raw data obtained after acquisition were FID. A Fourier transform was applied for each spectrum with a specific smoothing (efp with LB = 0.3 and SI = 256K). Phase and baseline correction were also performed using automatic tools form TopSpin 4.1.3 software before the manual integration of specific signals belonging to metabolites present in the samples. Absolute quantitation of metabolites of interest was performed using Sodium-2,2,3,3-trimethylsilylpropionate D4 (TSP-d4) as a reference external standard. The quality of the analysis is based on the good resolution of the spectrum. The acceptance criteria for this parameter is width at half height for TSP-d4 signal < 5Hz (complex medium).

*Bioenergetic investigations in human skin fibroblasts.* Overall, 7.5.10^5^ dermal fibroblasts were seeded in T175 flasks and 48h before the seahorse analysis the cells were treated with different sugar concentration as mentioned in the article. Five hours before the measure of respiration in the Seahorse XFe96 analyzer, the control and treated cells were seeded in XF 96-well cell culture microplates (Seahorse Bioscience) at 1.10^4^ cells/well in 160 μL of medium (#A144300, Gibco) complemented with 4 mM glutamine (Gibco), 1 mM pyruvate (Gibco) and 10 % fetal bovine serum (Gibco) and then incubated at 37 °C, 5 % CO_2_ for 5h until the total attachment of the cells. Measurement of endogenous respiratory rate was then performed as follows. The OCR and ECAR were measured following the seahorse running program detailed next: Injection port A = 1 μM Oligomycin, Injection port B = 1 μM CCCP, and injection port C = 0.5 μM Rotenone/Antimycin A. The number of cells was determined by using the Sulforhodamine B assay [10] at the end of the seahorse run protocol.

### 2.5 Enzyme-linked immunosorbent assay (ELISA)

Human Dermal Fibroblasts (HDF) were cultured in Dulbecco's Modified Eagle Medium (DMEM) supplemented with 10 % fetal bovine serum (FBS) and 1 % penicillin-streptomycin with 5.55 mM or 12 mM of glucose. The conditioned media was collected from the cultured HDF supernatant after 48 h. For detection of secreted GDF15, a commercially available enzyme-linked immunosorbent assay (ELISA) kit from Invitrogen was used (#EHGDF15). We carried out the dosage according to the manufacturer's instructions.

### 2.6 Cell and tissue imaging

*Live imaging of dermal fibroblasts*. Human Dermal Fibroblasts were seeded into Labteck II (Chamber Slide™ System 8 chambers, fisher Scientific) at 10 000 cells per chamber. Mitotracker Red (Invitrogen, #M22425) was incubated at 50 nM during 10 min in 5 % CO2 at 37 °C. Images were obtained by confocal microscopy (Leica DMI8) and analyzed using ImageJ software.

*Immunofluorescence of Human Reconstituted Skin.* HRS were embedded in Tissue-Tek O.C.T™ (Sakura) and snap-frozen in liquid nitrogen before storage at −80 °C. 10 μm sections were cut with a cryostat (Leica) on Superfrost slides and stored at −80 °C. The sections were dried for 20 min in a dry 37 °C incubator and then fixed in acetone at −20 °C before immunofluorescence staining. Primary antibody used were: Collagen 1(#20111-, Novotec) at 1/200; Collagen 6 (#2061, Novotec) at 1/1000; MTERF3 (#ab230232, Abcam) at 1/500; TOMM20 (#ab56783, Abcam) at 1/500; Involucrin (#sc-398221, Santa Cruz) at 1/200, MKI67 (#180192Z, Invitrogen) at 1/200 and Corneodesmosin (#ab204235, Abcam) at 1/1000. Nucleus were marked with DAPI (#D1306, Molecular Probes) at 0.6 μM. Secondary antibody used were: Goat Anti-Mouse AF568 (#A11004, Invitrogen) at 1/500; Chicken Anti-Rabbit AF647 (#A21443, Invitrogen) at 1/500; Goat Anti-Mouse AF647 (#A21235, Invitrogen) at 1/500 and Donkey Anti-Rabbit AF568 (#A10042, Invitrogen) at 1/2500. Slides were mounted in gelatin-containing medium for fluorescence (Dako) and stored at 4 °C before imaging. Images were obtained by confocal microscopy (Leica DMI8) and analyzed using ImageJ software. Fluorescent signal intensity on HRS was measured relative to the analyzed surface or number of nuclei, and an average of 10 fields analyzed per sample. An ANOVA statistical analysis was performed with the GraphPad software.

*Human Reconstituted Skin Histology Study.* After 24 h in buffered formalin, samples were dried and impregnated in paraffin, 10 μm sections were made and mounted on slides for histological studies. 3 explants per condition were prepared and stained using Masson's trichrome and Hematoxylin Eosin staining. The general morphology was evaluated by microscopy analysis (Leica optical microscope type DMLB) at the magnification of 40. 5 pictures per explants were taken using the VS120 Olympus Evident scanner. An ANOVA statistical analysis was performed with the GraphPad software.

### 2.7 Statistics analysis

All values were the mean ± SEM of minimal three independent experiments (biological replicates). The statistics were performed with GraphPad Prism 9 software using a 2-way unpaired Student's *t*-test to compare two independent groups or paired for sequential measurements. Evaluation of the gaussian distribution of the data was performed prior to use *t*-test or ANOVA. One-way and two-way ANOVA with Dunnett test correction were performed when comparing different groups (as precised in the legends). Statistical significance was determined at ∗P < 0.05, ∗∗P < 0.01, ∗∗∗P < 0.001.

### 2.8 Schematics and illustrations

All diagrams and illustrations were created with *BioRender.com*, an online scientific illustration software. Figures were generated using GraphPad Prism (version 10.2.3).

## 3 Results

### 3.1 Skin collagen network is altered by high (12 mM) glucose stress

**Fig. 1:**
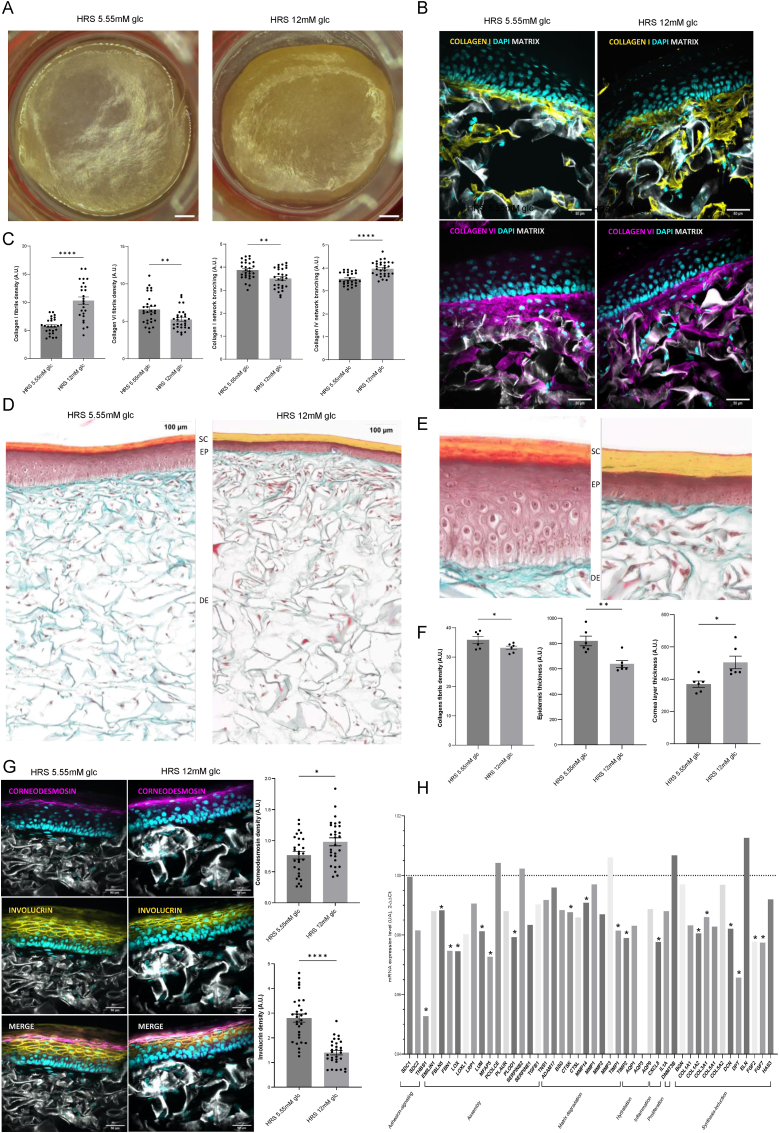
**Dermal collagen network and human skin reconstruction are altered by 12 mM glucose stress**. **A)** Macroscopic view of HRS cultivated 38 days in 5.55 mM or 12 mM glucose media. The images were acquired using a binocular microscope. Scale bar 0.6 cm. **B)** Immunofluorescence detection of collagens I and VI in HRS exposed to 5.55 mM or 12 mM glucose. **C**) Signal was quantified by measuring the fluorescence intensity of collagen normalized to nucleus number (as detected using DAPI staining). Collagen network morphology was analyzed by applying an internal deconvolution filter, then by counting the number of objects and relating them to the circularity parameter. Results are given as arbitrary units (A.U.). 10 images were acquired per HRS (N = 3). **D, E)** Masson trichrome staining of HRS exposed to 5.55 mM or 12 mM glucose. Images were acquired using a Scanner VS120 Olympus (Obj x10). Staining was performed for collagen fibers (blue-green), keratin fibers (red) and cell nuclei (dark red or purple). SC = stratum corneum; EP = epidermis; DE = dermis. **F**) Collagen density was quantified by measuring the staining intensity normalized to the nucleus number (DAPI staining) (N = 6). Quantification of epidermis and stratum corneum thickness was given as arbitrary units (A.U.) (N = 6) **G)** Immunofluorescence of corneodesmosine and involucrine in HRS exposed to 5.55 mM or 12 mM glucose. Markers expression was quantified by measuring the fluorescence intensity, as normalized by the number of nuclei (DAPI staining). 10 images were acquired per HRS (N = 3). **H,I**) Comparative Taqman Low Density Array (TLDA) real-time RT-PCR gene expression analyses at day 38. HRS cultivated in 12 mM glucose were compared to those grown in 5.5 mM glucose. N = 4.

We first investigated the impact of 12 mM glucose treatment on human skin organization. Observations using a binocular microscope did not show any marked difference between human reconstructed skin (HRS) reconstituted with 5.55 mM glucose or 12 mM glucose (Fig. 1A). However, confocal microscopy study of the dermal collagen network (Fig. 1B) showed (i) increased collagen I fiber density and (ii) greater fragmentation (Fig. 1B and C). Conversely, the collagen VI fiber density was reduced, and the corresponding network was less structured. Collagen type I is the primary structural component of the skin, providing strength and support to resist stretching and tearing, while collagen type VI forms a supportive network that enhances skin elasticity and hydration. Histology studies on HRS reconstituted with 5.55 mM or 12 mM glucose revealed a significant decrease in total collagen fibrils density under 12 mM glucose stress (Fig. 1D and E). Furthermore, epidermis thickness was significantly reduced in the HRS grown in 12 mM glucose, while the stratum corneum was greater (Fig. 1E and F). Moreover, the levels of two key markers of the stratum corneum, involucrin and corneodesmosin, were altered (Fig. 1G). Accordingly, Taqman low density array (TLDA) real-time RT‒qPCR experiments performed on (Fig. 1H and I) revealed the significant downregulation of matrix synthesis (COL1A2, COL3A1, DCN, DPT, FGF2 and FGF7) and matrix assembly (THBS1, FBLN5, FBN1, LOX, LUM, MFAP5 and PLOD1) components. The levels of matrix degradation and inflammation executors (CTSK, MMP14, TIMP1 and TIMP2) were also decreased. These findings indicate that 12 mM glucose stress alters skin physiology.

### 3.2 High-glucose stress represses the mitochondrial proteome and metabolome

**Fig. 2:**
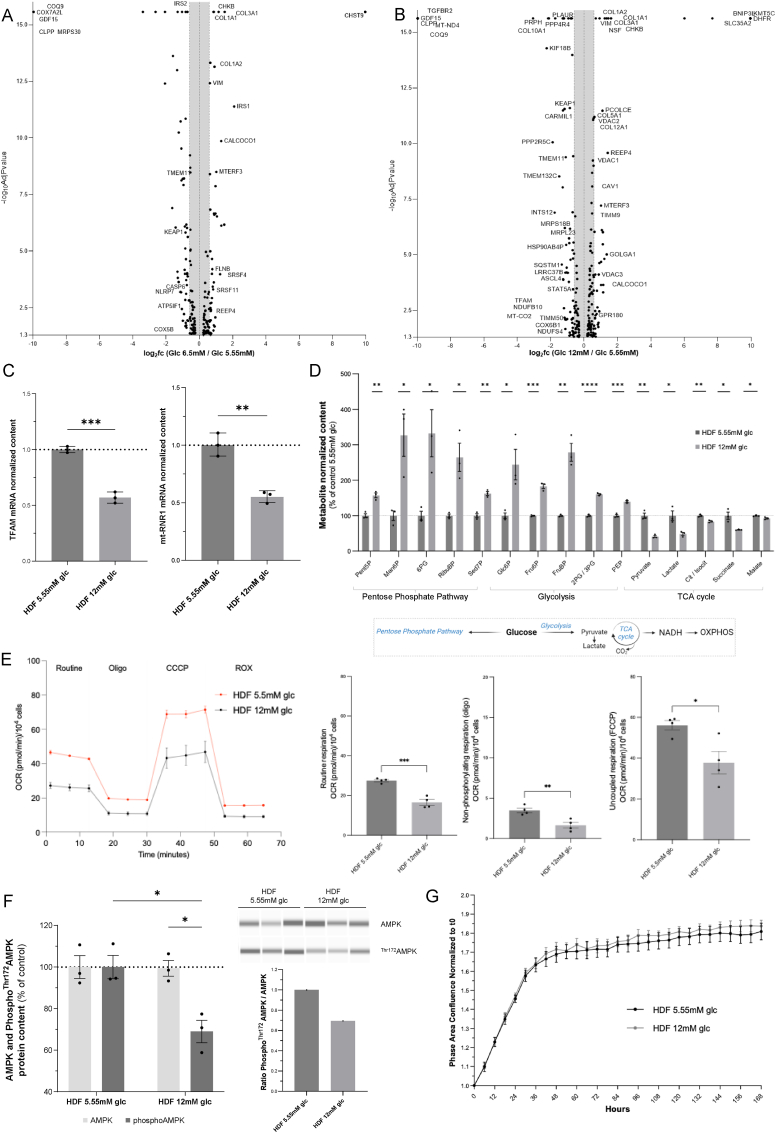
**High glucose stress represses mitochondrial proteome, metabolome and bioenergetics. A)** Label free unbiased differential proteomic study of HDF exposed 48H to 6.5 mM glucose as compared to 5.55 mM. The repressed proteins are shown on the left side of the Volcano Plot and the over-represented proteins on the right. The X axis gives the quantitative change expressed of log2 of the fold change (6.5 mM versus 5.55 mM). The Y axis provides the significancy of the changes expressed as the -log10 of the Adjusted P value (N = 4). **B)** Similar study was performed between the 12 mM and the 5.55 mM glucose conditions. **C)** Quantification of RNA transcripts by Taqman quantitative RTPCR of mt-RNR1 (Mitochondrially Encoded 12S RRNA) and TFAM (Transcription Factor A, Mitochondrial) in HDF grown in 5.55 mM or 12 mM glucose. Normalization of the data was performed to GusB (β-glucuronidase) (N = 3). **D)** Metabolomic analysis of HDF grown 48H in DMEM with 5.55 mM or 12 mM of glucose (N = 3). The following metabolites were quantified: Glucose-6-phosphate (Glc6P), Fructose-6-phosphate (Fru6P), Fructose-1.6-bisphosphate (FruBP), 3-phosphoglycerate and 2-phosphoglycerate (2PG/3 PG), phosphoenolpyruvate (PEP), pentose-5-phosphate (Pent5P), Mannone-6-phosphate, 6-phosphogluconate (6-PG), ribulose-1,5-bisphosphate (RibuBP), sedoheptulose-7-phosphate (Sed7P), pyruvate, lactate, citrate and isocitrate (Cit/Isocit), Succinate and Malate. The metabolite content was expressed as % of the 5.55 mM control conditions. **E)** Oxygen consumption rate (OCR) was measured using the Seahorse XFe96. Routine respiration, non-phosphorylating respiration (Oligomycin) and uncoupled respiration (CCCP) were determined in human skin fibroblasts grown during 48h in DMEM with 5.55 mM or 12 mM of glucose. Analysis was performed using the Wave 2.6 software (N = 6). The cell passage numbers used in the different experiments was between P6 and P8. **F)** Determination by Simple WES of AMPK and phospho-AMPK^Thr172^ protein content in HDF grown during 48H in DMEM with 5.55 mM or 12 mM of glucose. The ratio of phospho/total AMPK is given in panel F. Protein expression was normalized to total protein content (N = 3). **G)** Growth curves of HDF grown in DMEM with 5.55 mM or 12 mM of glucose (N = 12). All data are expressed as the mean ± SEM. ∗P < 0.05, ∗∗P < 0.01, ∗∗∗P < 0.001. Unpaired *t*-test was used for panels A,B,C,E and F. Regarding panel D, unpaired *t*-test was used for each metabolite, while comparing 5.55 mM and 12 mM glucose.

To elucidate the global cellular adaptive response to high-glucose stress, we performed an unsupervised study of the molecular changes detected at the proteome level (Fig. 2). This analysis revealed major changes at the mitochondrial proteome level, with significant repression of several mitochondrial proteins in response to either 6.5 mM or 12 mM glucose exposure (Table S1 and Table S2, respectively). Specifically, proteins essential for mitochondrial biogenesis and respiration, such as TFAM, MT-ND4, COQ9, MT-CO2, NDUFB10, COX6B1, TIMM50, CLPP and NDUFS4, were strongly repressed by 12 mM glucose stress, while the negative regulator of mitochondrial transcription and translation MTERF3 was overexpressed, as was the mitophagy activator BNIP3L (Tables S1 and S2). Further analysis of the non-mitochondrial proteins inhibited by 12 mM glucose stress revealed the redox regulator Keap1. The level of mitochondrial reactive oxygen species (ROS) determined *in situ* [12] was significantly higher in HDFs exposed to 25 mM glucose than in those exposed to 5.55 mM glucose, with no significant change observed in HDFs exposed to 12 mM glucose (Fig. S1). Finally, the expression of the cytokine GDF15 which can stimulate the OXPHOS activators AMPK and PGC1α and promote mitochondrial respiration [13,14], was strongly and highly significantly repressed (Fig. 2A and B) by both 6.5 mM and 12 mM glucose exposure. The quantity of TFAM, a key regulator of mitochondrial DNA replication and biogenesis, was also reduced by high-glucose stress, as was the mitochondrial DNA content (Fig. 2C). Glucose metabolism was analyzed in human dermal skin fibroblasts exposed to 12 mM glucose stress using IC-HRMS metabolomics (Fig. 2D). The results indicated a significant increase in the levels of most glycolysis and pentose phosphate pathway intermediates. Conversely, a significant decrease in pyruvate, lactate and TCA cycle metabolites (Fig. 2D), including citrate, isocitrate, succinate and malate, was also observed at steady-state under 12 mM glucose stress conditions. These findings led to the hypothesis that the quantity and/or activity of components of pyruvate oxidative metabolism, such as the TCA cycle and OXPHOS enzyme complexes, could be altered upon high-glucose stress exposure. Accordingly, a functional analysis of mitochondrial respiration revealed a reduction in oxidative phosphorylation in cells exposed to 12 mM glucose stress (Fig. 2E). The level of extracellular acidification rate (ECAR) which provides an indirect measure of the lactic acid derived from glycolysis (or of the CO_2_ generated by the TCA cycle [15]) was increased in these conditions (Fig. S2A). Also, the cells did not exhibit an energy crisis because the AMPK signaling pathway was not activated and was even suppressed under 12 mM glucose conditions (Fig. 2F), suggesting predominant glycolytic metabolism. Likewise, no significant change in cell proliferative activity was detected (Fig. 2G). Taken together, these data describe proteomic and metabolic responses to moderate (6.5 mM) and high (12 mM)-glucose stress and suggest mitochondrial repression as the specific mechanism of such responses.

### 3.3 GDF15 prevents high glucose stress toxicity on skin physiology

**Fig. 3:**
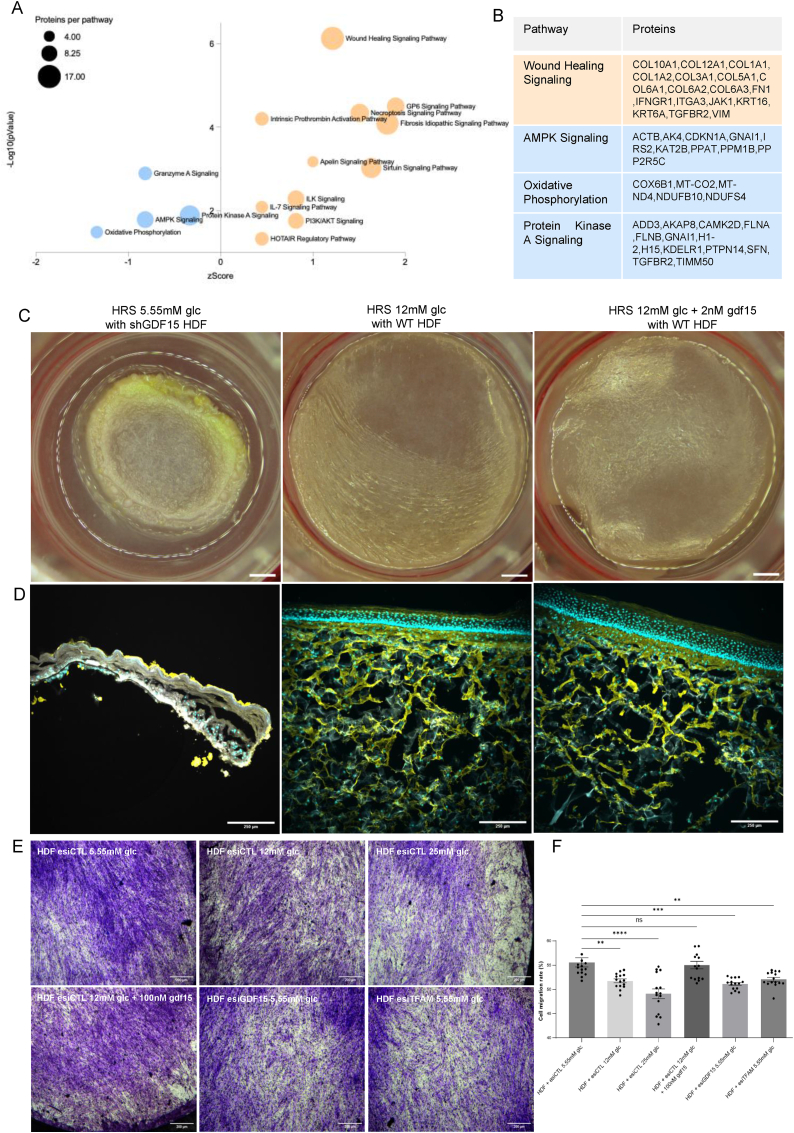
**GDF15 biosynthesis is indispensable for human skin reconstruction**. **A)** The effect of 100 nM GDF15 supplementation was determined using comparative proteomics on HDFs exposed to 12 mM glucose for 48H*.* The pathway analysis is shown as a bubble volcano plot (significant pathways with -logAdjPvalue>1.3 are shown. The pathways with blue dots are inhibited while pathways with orange dots are activated. The number of proteins detected for each pathway is represented by the diameter of each dot. Activation or inhibition was determined using the Z-score calculated by IPA Qiagen. **B)** Proteins of the Wound Healing Signaling, AMPK Signaling, Oxidative Phosphorylation or Protein Kinase A Signaling altered by the 12 mM glucose treatment are shown. **C**) Human skin reconstruction was performed using fiboblasts expressing a shGDF15, wild-type fibroblasts exposed to 12 mM glucose or wild-type fibroblasts exposed to 12 mM glucose and supplemented with 2 nM GDF15. development. Macroscopic view is shown with a scale bar of 0.6 cm, **D)** Immunofluorescence study of HRS using DAPI marker (blue), Collagen I (yellow) and MKi67 (pink). Scale bar. 50 μM, N = 3. **E**,**F**) Migration assay of HDF cultivated in 5.55 mM, 12 mM or 25 mM glucose and HDF cultivated in 12 mM glucose supplemented with 100 nM gdf15, HDF transfected with esiGDF15 or esiTFAM (N = 15). All data were expressed as the mean ± SEM. ∗P < 0.05, ∗∗P < 0.01, ∗∗∗P < 0.001. Ordinary one-way ANOVA with Dunett's test correction was used for panel F.

The proteome reprogramming of HDFs grown in 12 mM glucose (Fig. 2B) revealed modified expression of different types of collagens (COL1A2, COL3A1, COL1A1, COL5A1, COL12A1, COL6A2 and COL6A3) and the corresponding pathway analysis showed alteration of wound healing signaling, ILK signaling or GP6 signaling which are essential to skin physiology (Fig. 3A and B). Previous studies established a link between GDF15 expression and skin physiology [16] or GDF15 levels and energy metabolism [[17], [18], [19], [20], [21], [22]]. Here, we questioned the role of GDF15 on the dual modulation of these two processes. To investigate how GDF15 (strongly repressed by high-glucose; Fig. 2A and B) contributes to skin dermal structure alterations, we performed skin reconstruction using cells expressing shGDF15. The HRS produced from shGDF15 HDFs could not be properly reconstructed (Fig. 3C and D) and the HRS reconstituted in 12 mM glucose had an altered collagen network (Fig. 1, Fig. 3D). In contrast, 100 nM GDF15 supplementation reversed this phenotype and normalized the collagen network (Figs. S3A–H). Accordingly, a 3D migration assay of human skin fibroblasts showed that downregulating GDF15 expression using esiGDF15 significantly altered haptotaxis (Fig. 3E and F). Similar findings were obtained using a siRNA targeting TFAM (Fig. 3E and F). These findings indicate that high-glucose stress, GDF15 downregulation and TFAM-dependent OXPHOS inhibition alter skin integrity at the level of the dermis and epidermis. To study the mechanisms involved in the rescuing effect of GDF15 supplementation on high glucose stress exposure, we performed the unsupervised analysis of human skin fibroblasts proteome. The results (Table S3) showed that GDF15 supplementation promoted a significant increase in proteins involved in mitochondrial energy transduction including glutaminase, mitochondrial ribosome (MRPS17), mitochondrial ATP synthesis (ATP5F1EP2), complex IV assembly (COX7A2L), antioxydant defenses (GSTT1, GSTM3 and GSTP1) and glycolysis (hexokinase 2, PGK1). Moreover, this analysis identified an increase in AMPK beta subunit (PRKAB1), in agreement with studies showing that GDF15 activates AMPK signaling [17,18,21]. At last, the pathway analysis study of the rescuing effect of GDF15 on high glucose stress exposure showed the activation of ‘Epithelial Adherens Junctions’ and of ‘Gap Junctions’ signaling (Table S4), suggesting changes in short-range messaging between skin cells. An effect of GDF15 was also observed at the level of the mitochondrial proteome organization (Fig. S4). Mitochondrial network density, tubule length and network branching were increased by GDF15 supplementation (Fig. S4).

### 3.4 High-glucose stress activates the mitochondrial repressor MTERF3

**Fig. 4:**
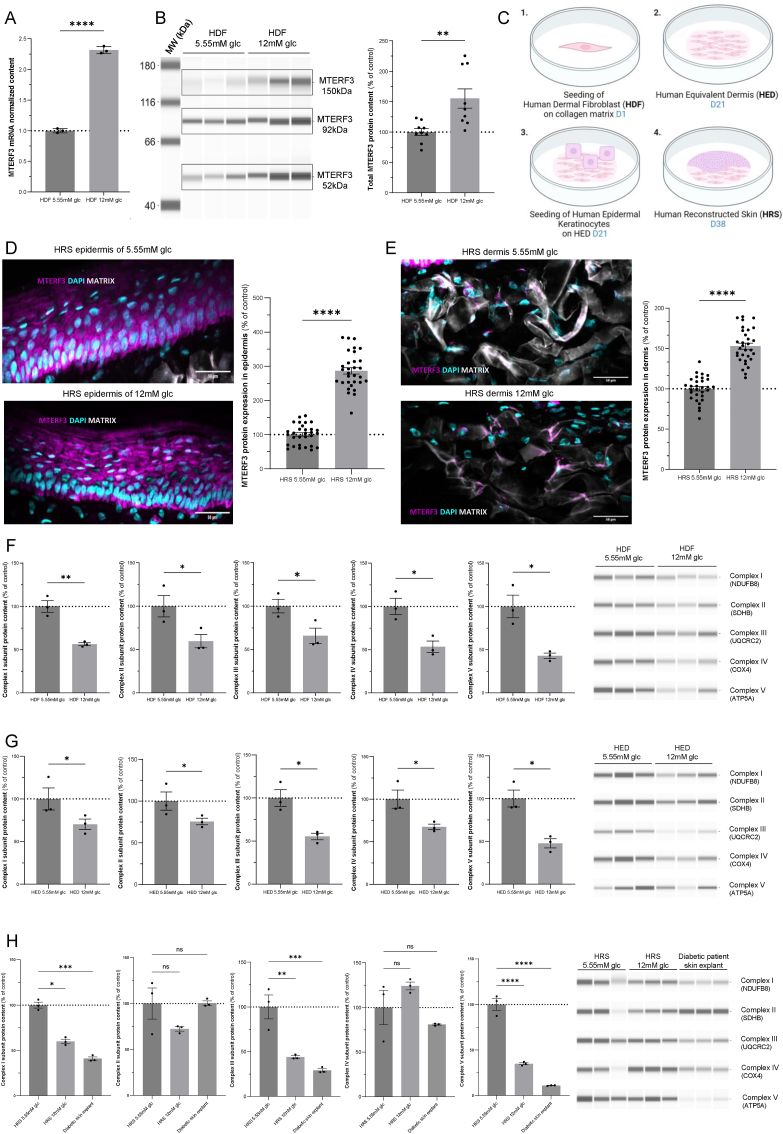
**High glucose stress activates the mitochondrial repressor MTERF3. A)** Quantification of the MTERF3 (Mitochondrial Transcription Termination Factor 3) RNA transcript by taqman quantitative PCR in HDF grown in 5.55 mM or 12 mM glucose. Normalization of the data was performed using GusB (β-glucuronidase) (N = 3). **B)** Determination by Simple WES of MTERF3 protein expression in HDF cultivated 48H in DMEM with 5.55 mM or 12 mM glucose. Total protein was used for normalization. (N = 3). **C)** Human Reconstructed Skin (HRS) generation process. HDF were seeded on bovine collagen matrix. After 21 days, Human Equivalent Dermis (HED) was produced. Human Epidermal Keratinocytes were seeded on HED. After 38 days, HRS was produced. **D)** Immunofluorescence analysis of MTERF3 expression in HRS epidermis grown in 5.55 mM or 12 mM glucose. Signal intensity was quantified by measuring MTERF3 fluorescence (purple color) normalized to the number of cells, as determined by the number of nuclei (DAPI staining; blue color). 10 images were acquired per HRS (N = 3). **E)** Similar analysis was performed in HRS dermis. **F)** Determination by Simple WES of the protein expression level of various mitochondrial respiratory chain subunits in human dermal fibroblasts (HDF): complex I (NADH:Ubiquinone Oxidoreductase Subunit B8), complex II (Succinate dehydrogenase [ubiquinone] iron-sulfur subunit), complex III (Ubiquinol-Cyt C Reductase Core Protein 2), complex IV (Cyt C Oxidase Subunit 4) and complex V (ATP synthase alpha subunit) in HDF cultivated 48 h in DMEM with 5.55 mM or 12 mM of glucose. Total protein loading was used for normalization (N = 3). Similar analysis was performed in **G)** Human Equivalent Dermis (HED, N = 3) and **H)** Human Reconstructed Skin (HRS, N = 3). All data are expressed as the mean ± SEM. ∗P < 0.05, ∗∗P < 0.01, ∗∗∗P < 0.001. Unpaired *t*-test was for all panels.

The activation of MTERF3 by mild (6.5 mM) or high (12 mM) glucose stress (Fig. 2A and B) was further studied using a variety of genetic and biochemical methods. First, MTERF3 gene expression analysis by Taqman RT-qPCR revealed 2-fold significant (pvalue <0.0001) activation in human dermal skin fibroblasts exposed to 12 mM glucose (Fig. 4A). Accordingly, an MTERF3 protein expression assay using automated capillary electrophoresis western analysis (WES) also revealed a significant increase (Fig. 4B). Three forms of MTERF3 were identified by WES at 52, 92 and 150 kDa (apparent molecular weight), with all exhibiting increased protein content (Fig. 4B). MTERF3 expression was then investigated in human reconstructed skin using immunohistochemistry methods (Fig. 4C). To gain further insight into skin physiology, we generated human reconstructed skin (HRS) by producing human equivalent dermis (HED) from human dermal fibroblasts (HDFs) and human epidermal keratinocytes (Fig. 4C). MTERF3 was significantly increased both in the epidermis (Fig. 4D) and dermis (Fig. 4E) when the skin was reconstructed in 12 mM glucose medium. MTERF3 was previously characterized as a suppressor of mitochondrial gene expression but also as a negative regulator of mitochondrial protein translation [[23], [24], [25], [26]]. Accordingly, the expression of the respiratory chain proteins NDUFB8 (complex I), SDHB (complex II), UQCRC2 (complex III), COX4 (complex IV) and ATP5A (complex V) was significantly lower (p < 0.05) in HDFs exposed to 12 mM glucose than in those exposed to 5.55 mM glucose (Fig. 4F). Similar findings were obtained in HED (Fig. 4G). Finally, we analyzed respiratory chain complex I–V expression in the HRS and skin explants from diabetic patients (Fig. 4G). A strong and significant reduction in NDUFB8, UQCRC2 and ATP5A was observed compared to control. These findings confirm that high-glucose stress promotes MTERF3 activation and OXPHOS gene repression in the human dermis. Unsupervised proteomic analysis of the 12 mM glucose stress response also revealed an increase in the mitophagy regulator BNIP3L (Fig. 2). Accordingly, the *in situ* evaluation of mitophagy levels using the mito-Keima biosensor [27] revealed an increase in bulk-mitochondrial degradation following 12 mM glucose stress. Significant mitophagy induction was further observed following 25 mM glucose exposure (Fig. S5). These findings confirm that 12 mM glucose stress promotes mitophagy and MTERF3 activation which represses OXPHOS gene and protein expression in HDFs, HED and HRS. Higher glucose stress (25 mM) activated ROS generation and BNIP3L-dependent mitophagy stimulation.

### 3.5 MTERF3 ectopic overexpression represses OXPHOS

**Fig. 5:**
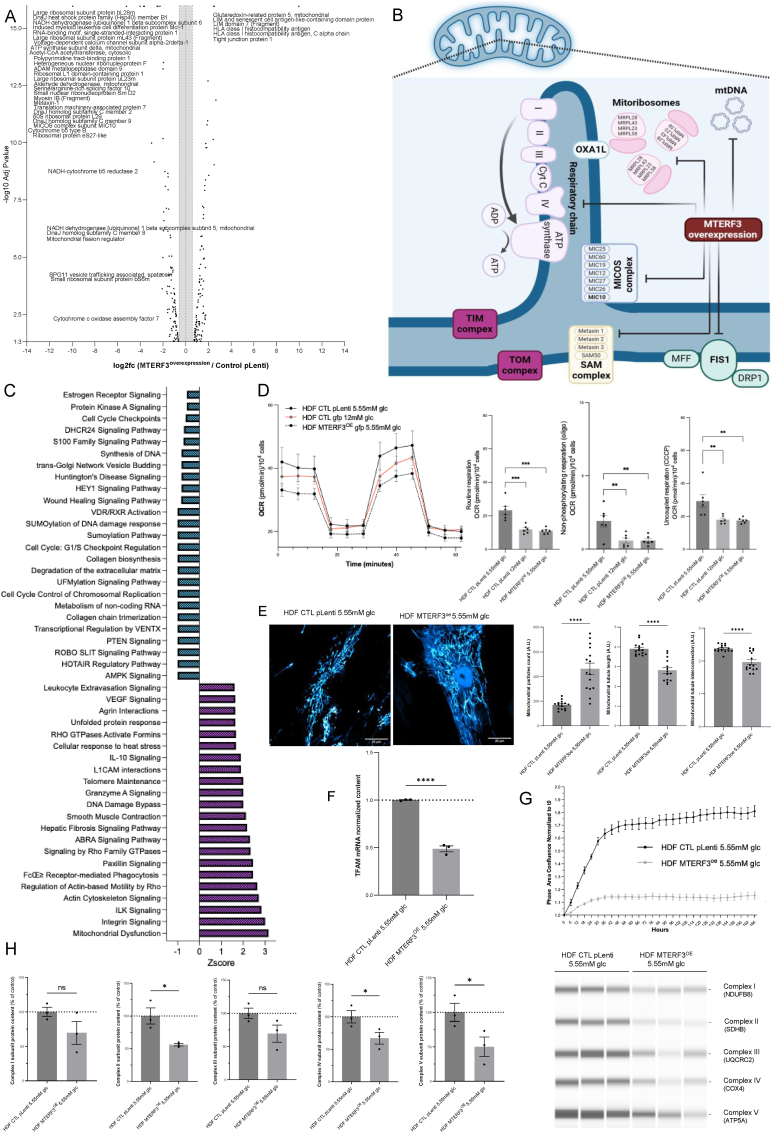
**MTERF3 ectopic overexpression represses OXPHOS. A)** Proteomic study of HDF overexpressing human MTERF3 (MTERF3OE) as compared to wild-type control expressing the empty plasmid *p*-lenti (N = 4). **B)** Schematic representation of the mitochondrial proteins reduced in expression (with Adjp<0.05) as a result of the MTERF3 overexpression (MTERF3OE) in HDF. **C)** Ingenuity Pathway Analysis (Z-score) of the proteins significantly altered in expression, in response to MTERF3 overexpression. **D)** Oxygen consumption rate (OCR) was measured using the Seahorse XFe96. Routine respiration, non-phosphorylating respiration (oligomycin) and uncoupled respiration (CCCP) were determined in human skin fibroblasts with MTERF3 overexpression, as compared to control cells grown in 5.55 mM or 12 mM of glucose (N = 6). **E)** Mitochondrial morphology study of HDF overexpressing MTERF3 and control cells grown in DMEM with 5.55 mM. Mitochondrial staining was performed using 50 nM of MitoTracker Red (N = 15). Three parameters of the mitochondrial network were analyzed using Image J: particles count, tubules length and interconnections. **F)** Quantification of TFAM (Transcription Factor A, Mitochondrial) RNA transcript by Taqman quantitative PCR in HDF overexpressing MTERF3, as compared to plenti-control cells. Normalization of the RT-QPCR data was performed to GusB (β-glucuronidase), N = 3. **G)** Growth curves of HDF lenti-control and MTERF3 overexpression grown during 7 days in DMEM with 5.55 mM or 12 mM of glucose (N = 12). **H)** Determination by Simple WES of the protein expression level of mitochondrial respiratory chain subunits: complex I (NADH:Ubiquinone Oxidoreductase Subunit B8), complex II (Succinate dehydrogenase [ubiquinone] iron-sulfur subunit), complex III (Ubiquinol-Cyt C Reductase Core Protein 2), complex IV (Cyt C Oxidase Subunit 4) and complex V (ATP synthase alpha subunit) in HDF control and MTERF3 overexpression cultivated 48 h in DMEM with 5.55 mM or 12 mM of glucose. Protein normalization was performed using total proteins (N = 3). All data are expressed as the mean ± SEM. ∗P < 0.05, ∗∗P < 0.01, ∗∗∗P < 0.001. Unpaired *t*-test was used for panels A,E,F and H.

To verify the causal relationship between MTERF3 and OXPHOS protein repression, human dermal skin fibroblasts overexpressing MTERF3 were generated (Fig. S6). The unbiased proteomic analysis of MTERF3-overexpressing cells revealed a strong and significant (AdjPvalue<0.05) decrease in the expression of many mitochondrial proteins involved in mitochondrial respiration and translation (Fig. 5A and B; Table S5). The unsupervised analysis of the signaling pathways altered by MTERF3 overexpression revealed the greatest changes in the Z scores for ‘mitochondrial dysfunction’ and ‘integrin signaling’. Negative changes in the Z scores were observed for ‘AMPK signaling’, ‘collagen chain trimerization’ or ‘wound healing signaling’, suggesting that MTERF3 could play a role in skin properties (Fig. 5C). The functional bioenergetic evaluation of the mitochondria showed that MTERF3 overexpression triggered a significant reduction in cell respiration (Fig. 5D) with no significant change in ECAR (Fig. S2A). The mitochondrial network morphology was also altered following MTERF3 overexpression (Fig. 5E). Specifically, mitochondrial network fragmentation was observed, as indicated by a greater number of particles with reduced length and decreased interconnections (Fig. 5E). Finally, the expression level of TFAM was reduced in MTERF3-overexpressing cells (Fig. 5F), and the proliferative activity of the cells also decreased (Fig. 5G). Moreover, the expression of the respiratory chain proteins SDHB (complex II), COX4 (complex IV) and ATP5A (complex V) was significantly lower in HDFs overexpressing MTERF3 than in HDFs under 5.55 mM glucose (Fig. 5H). These findings indicate that MTERF3 overexpression represses the mitochondrial proteome and function in human dermal skin fibroblasts.

### 3.6 NR2F2 glucose-dependent transcription factor upregulates MTERF3

**Fig. 6:**
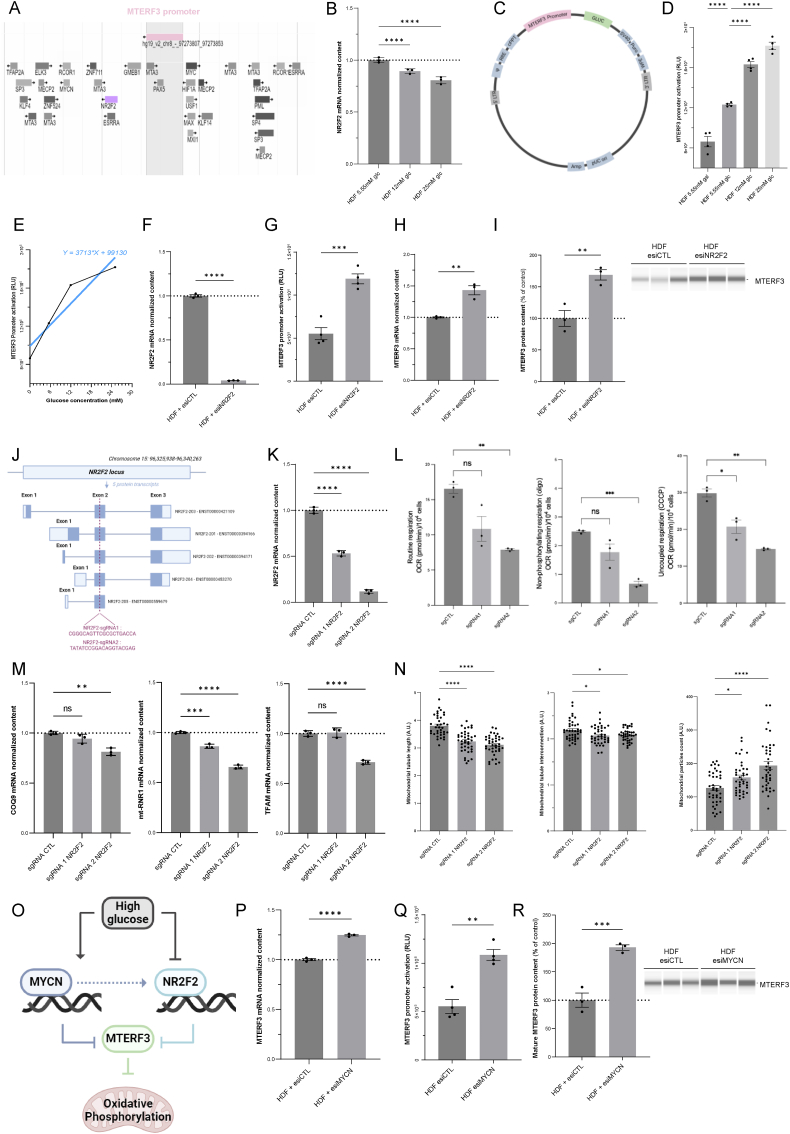
**NR2F2/Coup-TFII glucose-dependent transcription factor inhibition upregulates MTERF3. A)** Promoter region of the human MTERF3 gene and regulatory sites for transcription factors binding. Identification of NR2F2 binding site using Swiss Regulon (Expasy). **B)** Quantification of NR2F2 (Nuclear Receptor Subfamily 2 Group F Member 2) RNA transcript by taqman quantitative PCR in HDF cultivated in DMEM with 5.55 mM, 12 mM or 25 mM of glucose. Normalization of the data was performed to GusB (β-glucuronidase) (N = 3). **C)** MTERF3 promoter reporter activity assay using a Gaussia-luciferase (Gluc) lentiviral approach. **D)** Determination by bioluminescence of the MTERF3 promoter activity in HDF with lentiviral transduction of the reporter. Fibroblasts were grown 24 h in DMEM with 5.55 mM,12 mM or 25 mM of glucose, or 5.55 mM of galactose (N = 4). Data were normalized to the number of cells. **E)** Dose-dependent relationships between MTERF3 promoter activation and glucose concentration in the cell culture medium. **F)** Quantification of NR2F2 mRNA transcript by taqman quantitative PCR in HDF transfected with esiControl and esiNR2F2. Normalization of the data to GusB (β-glucuronidase), N = 3. **G)** MTERF3 promoter activity determination in HDF transfected with esiNR2F2 (N = 4). **H)** Quantification of MTERF3 mRNA transcript by taqman quantitative PCR in HDF transfected with esiNR2R2. Normalization of data to GusB (β-glucuronidase), N = 3. **I)** Determination by Simple WES of MTERF3 protein expression level in HDF transfected with esiNR2F2. Protein normalization was performed to total protein loading (N = 3). **J)** Schematic representation of lentiviral CRISPR guide RNA for the generation of NR2F2 knock-out. **K)** Quantification of NR2F2 mRNA transcripts by taqman quantitative PCR in stable HDF using sgControl, sgRNA 1 targeting NR2F2 and sgRNA2 targeting NR2F2. Normalization of the data to GusB (β-glucuronidase), N = 3. **L)** Oxygen consumption rate (OCR) was measured using the Seahorse XFe96. Routine respiration, non-phosphorylating respiration (oligo) and uncoupled respiration (CCCP) were determined in HDF expressing sgControl, sgRNA 1 targeting NR2F2 and sgRNA2 targeting NR2F2. **M)** Quantification of RNA transcripts by taqman quantitative PCR of COQ9 (coenzyme Q9), TFAM (Transcription Factor A, Mitochondrial) and MT-RNR1 (Mitochondrially Encoded 12S RRNA), in stable HDF sgControl, sgRNA 1 targeting NR2F2 and sgRNA2 targeting NR2F2. Normalization of data to GusB (β-glucuronidase), N = 3. **N)** Mitochondrial morphology of HDF expressing sgControl, sgRNA 1 targeting NR2F2 and sgRNA2 targeting NR2F2. Imaging was performed using 50 nM of MitoTracker Red (N = 40). **O)** Schematic representation of the impact of high(12 mM)-glucose medium on NR2F2, MYCN, MTERF3 and OXPHOS. **P)** Quantification of MTERF3 mRNA transcript by taqman quantitative PCR in HDF transfected with esiMYCN. Normalization of the data to GusB (β-glucuronidase), N = 3. **Q)** MTERF3 promoter activity assay in HDF transfected with esiMYCN (N = 4). **R)** Determination by Simple WES of the MTERF3 protein expression level in HDF transfected with esiMYCN (N = 4). All data were expressed as the mean ± SEM. ∗P < 0.05, ∗∗P < 0.01, ∗∗∗P < 0.001. Ordinary one-way ANOVA with Dunett's test correction was used for panel B, D, K, L, M and N. Unpaired *t*-test was used for panels F, G, H, I, P, Q and R.

To elucidate the molecular mechanisms underlying the glucose-dependent activation of MTERF3, we investigated the role of selected transcription regulators based on a survey of the MTERF3 gene promoter (Fig. 6A) [28]. More specifically, one transcription factor sensitive to glucose levels was identified: NR2F2/COUPTF-II [29,30]. Accordingly, we observed a dose-dependent reduction in NR2F2 expression upon the addition of 12 mM glucose to the medium (Fig. 6B). Using an MTERF3 promoter luminescent reporter assay, we observed that 12 mM glucose stress promoted the activation of MTERF3 transcription (Fig. 6C and D). This increase was proportional to the glucose concentration in the medium (Fig. 6E). The genetic inactivation of NR2F2 using siRNAs (Fig. 6F) triggered a significant increase in MTERF3 expression at the *(i)* promoter activation (Fig. 6G), *(ii)* mRNA (Fig. 6H) and *(iii)* protein content (Fig. 6I) levels. To further study the role of NR2F2 downregulation-mediated inhibition of OXPHOS, we generated a knockout construct in HDFs (Fig. 6J) by introducing a mutation affecting all NR2F2 transcript variants [31]. Knockout was achieved using two different single guide RNAs (sgRNAs), both targeting exon 2 of the NR2F2 gene. The first sgRNA (sgRNA 1) caused a decrease in almost 50 % of the mRNAs encoding NR2F2, and the second sgRNA (sgRNA 2) caused a decrease in more than 90 % of the mRNAs encoding NR2F2 (Fig. 6K). The bioenergetic study of NR2F2 knockout revealed significant gene dose-dependent inhibition of mitochondrial respiration (Fig. 6L) without any change in ECAR (Fig. S2D). Accordingly, NR2F2 sgRNA2-knockout HDFs showed a significant decrease in the levels of selected mitochondrial mRNAs (Fig. 6M). Finally, mitochondrial network fragmentation was also induced by NR2F2 knockout (Fig. 6N). These findings indicate that NR2F2 inhibition promotes MTERF3 activation and subsequent OXPHOS repression in response to 12 mM glucose stress (Fig. 6O). In addition to NR2F2, we also studied the possible regulation of OXPHOS repression by another glucose-dependent transcriptional regulator found in the MTERF3 promoter: MYCN. The genetic inactivation of MYCN using siRNAs triggered a significant increase in MTERF3 expression at the levels of (i) promoter activation (Fig. 6P), (ii) mRNA (Fig. 6Q) and (iii) protein content (Fig. 6R). MYCN downregulation using siRNAs also reduced the expression level of NR2F2, GDF15 and PGC1α (Figs. S7A–C). These findings indicate that NR2F2 and MYCN could cooperate to regulate MTERF3 and OXPHOS capacity in response to high (12 mM)-glucose stress.

### 3.7 High glucose stress represses GDF15 in the human dermis

**Fig. 7:**
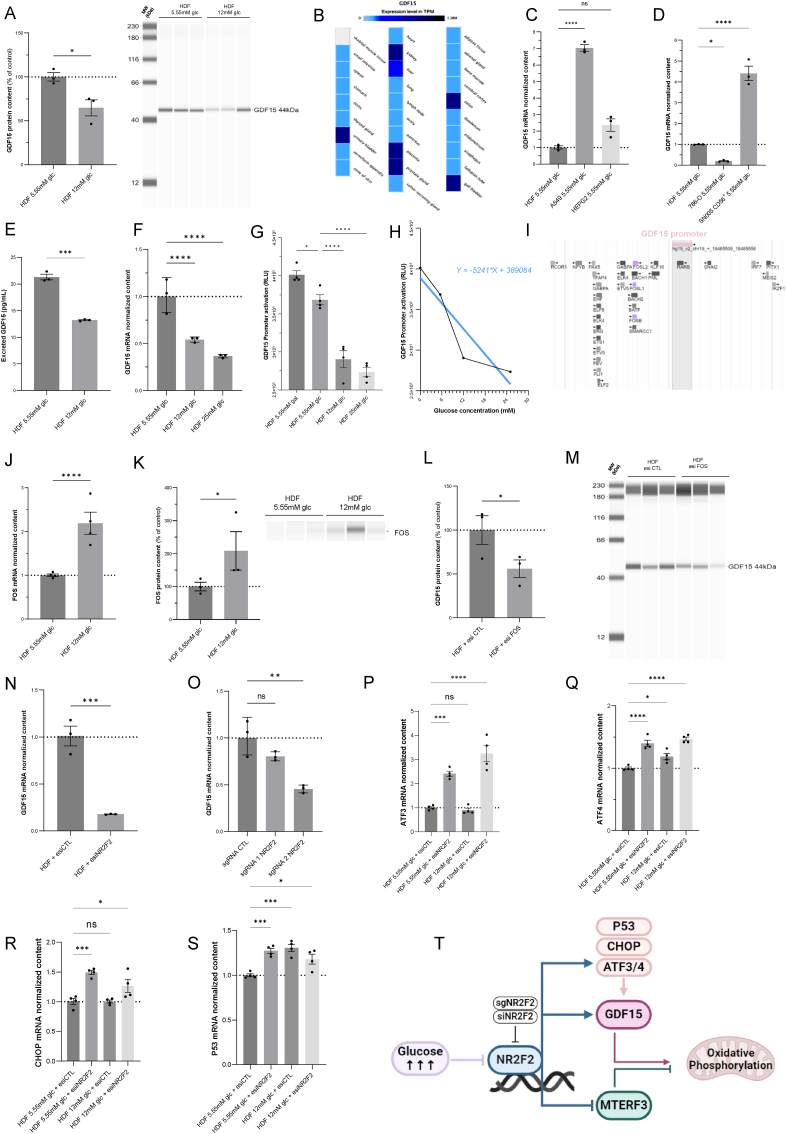
**cFOS and NR2F2 transcription factors mediate glucose-dependent repression of GDF15 in human dermis. A)** Determination by Simple WES of the protein expression level of proGDF15 in wild-type HDF expressing shcontrol or shGDF15 (N = 3). **B)** Expression of GDF15 in the skin from human protein expression atlas from EMBL-EBI (https://www.ebi.ac.uk) which includes RNA-seq analyses from tissue samples of 122 human individuals, representing 32 different tissues. The results are expressed as TPM (Transcripts Per Kilobase Million). **C)** Quantification of GDF15 mRNA transcripts by taqman quantitative PCR in HDF, A549 and HEPG2 (N = 3). **D)** Quantification of GDF15 mRNA transcripts by taqman quantitative PCR in HDF, 786-O and SN005 cells (N = 3). **E)** ELISA-Based Quantification of GDF15 secretion in HDFs. HDFs were cultured under conditions of normal (5.5 mM) and high (12 mM) glucose concentrations during 48h. GDF15 levels in the culture supernatants were quantified using an enzyme-linked immunosorbent assay (ELISA) following the manufacturer's instructions. **F)** Quantification of GDF15 mRNA transcripts by taqman quantitative PCR in HDF grown in 5.55 mM, 12 mM or 25 mM glucose (N = 3). **G)** GDF15 promoter activity in HDF grown 24 h in DMEM with 5.55 mM,12 mM or 25 mM of glucose or 5.55 mM of galactose (N = 4). **H)** Dose-dependent relationship between GDF15 promoter activation and glucose concentration in the medium. **I)** GDF15 gene promoter sequence with identification of the binding site for the FOS transcription factor (Swiss Regulon Expasy). **J)** Quantification of FOS mRNA transcript by taqman quantitative PCR in HDF cultivated in 5.55 mM or 12 mM glucose (N = 3). **K)** Determination by Simple WES of FOS protein expression level in HDF cultivated in 5.55 mM or 12 mM glucose (N = 3). **L-M)** Determination by Simple WES of GDF15 protein expression level in HDF transfected with esiFOS (N = 3). **N)** Quantification of GDF15 mRNA transcript by taqman quantitative PCR in HDF transfected with esiNR2F2 (N = 3). **O)** Quantification of GDF15 mRNA transcript by taqman quantitative PCR in HDF expressing sgControl, sgRNA 1 targeting NR2F2 and sgRNA2 targeting NR2F2. Normalization of the data to GusB (β-glucuronidase), N = 3. **P–S)** Quantification of ATF3, ATF4, CHOP and P53 mRNA transcripts by taqman quantitative PCR in HDF expressing siCTRL and esiNR2F2 in 5.5 mM glucose or 12 mM glucose growth medium. Normalization of the data to GusB (β-glucuronidase), N = 3. **T)** Schematic representation of the NR2F2-MTERF3-GDF15 axis and its control on OXPHOS function in response to glucose stress. All data are expressed as the mean ± SEM. ∗P < 0.05, ∗∗P < 0.01, ∗∗∗P < 0.001. Ordinary one-way ANOVA with Dunett's test correction was used for panel C, E and N. Unpaired *t*-test was used for panels A, B, H, I, J, K, L and M.

A proteomic study of human skin fibroblasts exposed to high glucose revealed a strong and highly significant decrease in GDF15 at both 6.5 mM and 12 mM glucose (Fig. 2A and B). The results of protein expression analyses using WES technology agreed with these findings (Fig. 7A). Validation of the WES methods for GDF15 quantification and comparison with conventional Western blot techniques (WB) was detailed in (Figs. S8A–D), using pure recombinant GDF15. Little is known on the expression of GDF15 in the skin so we performed a search using the human protein expression atlas from EMBL-EBI (https://www.ebi.ac.uk). The results (Fig. 7B) indicated that GDF15 expression in the skin is in the lowest range, as compared to tissues with high expression (eg. liver or kidney). Still the expression level of GDF15 mRNA in the skin was similar to what is found in the heart and the effect of GDF15 on heart physiology and pathology are well described [32,33], in contrast with the skin. The *in vitro* analysis of GDF15 expression in five human cell lines (Fig. 7C and D) also showed that HDFs have the lowest expression level of GDF15 mRNA. The entire gels of the different GDF15 WES experiments are shown in (Fig. S9).

Little is known on the GDF15 receptor GFRAL expression in human skin. The standardized data (ENSG00000187871.2) from The Genotype-Tissue Expression (GTEx) Portal detected significant GFRAL mRNA expression in the skin (Fig. S10A). Single cell RNAseq study (ENSG00000187871.2) also showed significant expression of GFRAL mRNA in human fibroblasts (Fig. S10B). Lastly, the ArchS4 database [34] showed significant expression of GFRAL mRNA in the skin (Fig. S10C). Still, GFRAL functions primarily in the CNS to mediate GDF15 signaling, with little to no significant protein expression reported in peripheral tissues. The expression pattern of GFRAL protein was recently reevaluated in healthy mice by utilizing immunofluorescence labeling methods and this work demonstrated that GFRAL-immunoreactivity (IR) was found in peripheral tissues [35]. Still, biochemical research using proteomics (whole tissue or single-cell) would be necessary to clarify whether truly functional GFRAL protein exists outside the central nervous system.

To further explore the variation of GDF15 expression in physiology we performed ELISA analyses on the secretome of human reconstructed skin (HRS) exposed to high glucose stress. The results showed a reduced secretion of GDF15 (Fig. 7E). Accordingly, high-glucose stress reduced GDF15 mRNA levels in human skin fibroblasts, as determined by Taqman RT‒qPCR in cells exposed to 12 mM and 25 mM glucose (Fig. 7F). To further study GDF15 transcriptional regulation in response to high-glucose stress, we generated a luminescent reporter of GDF15 promoter activation. This approach revealed a significant and dose-dependent decrease in GDF15 expression in response to the addition of 0 mM–25 mM glucose (Fig. 7G and H). These findings suggest that GDF15 could act as a messenger of glucose concentration in the dermis.

### 3.8 Multifactorial regulation of GDF15 in high-glucose conditions

GDF15 harbors three transcription factor-binding sites for FOS members (Fig. 7I), and FOS is a glucose-dependent transcription factor [36]. In our study, FOS expression was increased in HDFs exposed to 12 mM glucose (Fig. 7J and K). siRNAs targeting FOS inhibited GDF15 expression under high glucose stress conditions at the protein content level (Fig. 7L and M). These findings could suggest that FOS positively regulates GDF15 transcription. Moreover, genetic studies of NR2F2 targets (ENCODE transcription factor ChIP-seq database, encodeproject.org, Stanford University) identified binding sites on the GDF15 locus. Accordingly, the use of an siRNA targeting NR2F2 reduced GDF15 mRNA expression (Fig. 7N). Similarly, NR2F2 sgRNA2-knockout HDFs also showed a reduction in GDF15 transcript content (Fig. 7O). These data indicate that NR2F2 positively regulates GDF15. Previous studies showed that GDF15 is regulated by many transcription factors, such as ATF3 and 4, CHOP and p53 [32]. Therefore, we measured the expression level of these factors in response to high glucose stress, and following NR2F2 downregulation with siRNA (Fig. 7P–S). The results showed that NR2F2 genetic inhibition activated ATF3, ATF4 and CHOP, suggesting an antagonistic relationship between these factors concerning the regulation of GDF15 under high glucose stress. Indeed, NR2F2 inhibition by high glucose triggered a reduction of GDF15 expression, while ATF3/4 and CHOP which promote GDF15 expression were stimulated by NR2F2 inhibition. These findings could support the idea that the regulation of GDF15 under high glucose conditions is multifactorial (Fig. 7T).

### 3.9 GDF15 downregulation represses mitochondrial biogenesis

**Fig. 8:**
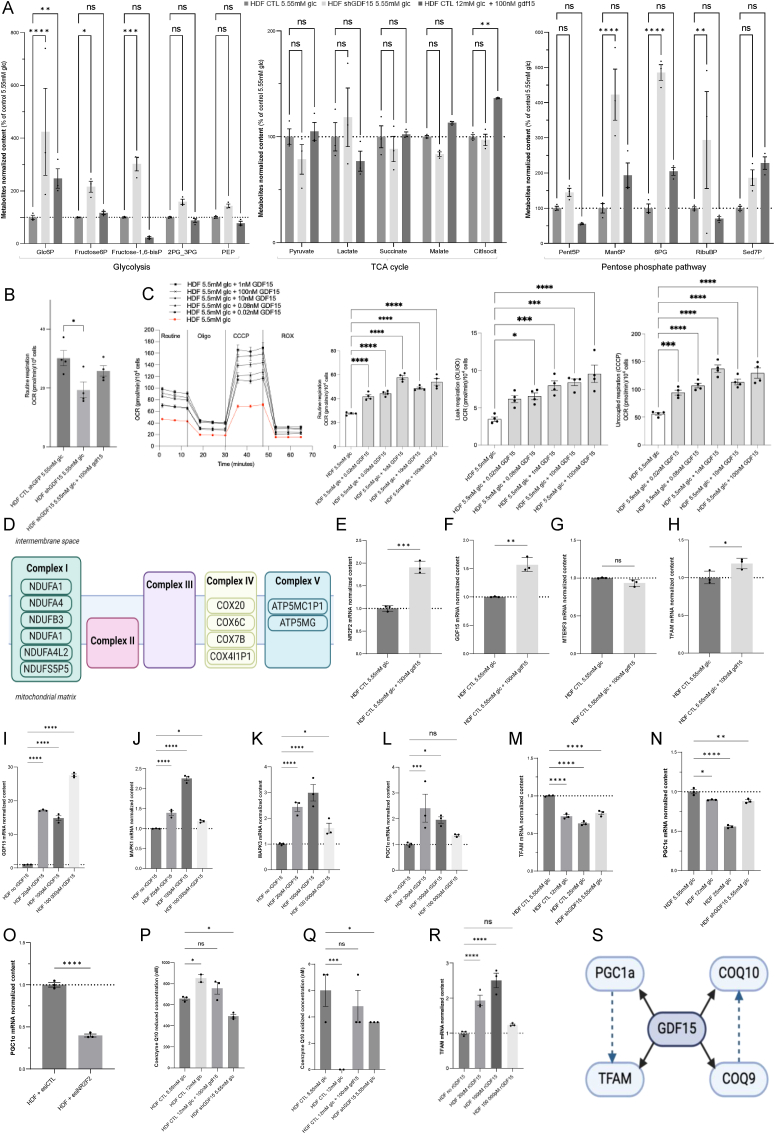
**GDF15 inhibition by hyperglycemia or shRNA alters mitochondrial biogenesis. A)** Metabolomic profile of HDF grown 48H in DMEM with 5.55 mM or 12 mM + 100 nM GDF15 of glucose and HDF expressing a shGDF15 cultivated in 5.55 mM glucose (N = 3). **B)** Oxygen consumption rate (OCR) was measured using the Seahorse XFe96. Routine respiration and uncoupled respiration (CCCP) were determined in HDF grown in 5.55 mM glucose and HDF expressing shGDF15 grown in 5.55 mM glucose or 5.55 mM glucose supplemented with 100 nM gdf15. **C)** Oxygen consumption rate (OCR) was measured using the Seahorse XFe96. Routine respiration and uncoupled respiration (CCCP) were determined in HDF grown in 5.55 mM glucose and supplemented with low doses of gdf15: 20pM, 80pM, 1 nM, 10 nM and 100 nM. **D)** Mitochondrial respiratory chain proteins (gene loci) specifically activated at the level of chromatin accessibility by GDF15 100 nM. **E-H)** Quantification of mRNA transcripts by taqman quantitative PCR for NR2F2, GDF15, MTERF3 and TFAM in HDF cultivated with 5.55 mM glucose or 5.55 mM glucose supplemented with 100 nM gdf15. Normalization of the data was performed to GusB (β-glucuronidase), N = 3. **I-L)** Quantification of mRNA transcripts by taqman quantitative PCR for GDF15, MAPK1, MAPK3, PGC1α (Peroxisome proliferator-activated receptor-gamma coactivator 1 alpha) in HDF cultivated with 5.55 mM glucose supplemented with low doses of gdf15. Normalization of the data was performed to GusB (β-glucuronidase), N = 3 **M)** Quantification of TFAM mRNA transcripts by taqman quantitative PCR in HDF cultivated with 5.55 mM, 12 mM or 25 mM glucose or in HDF expressing shGDF15 grown in 5.55 mM glucose. Normalization of data was performed to GusB (β-glucuronidase), N = 3. **N)** Quantification of PGC1α mRNA transcript by taqman quantitative PCR in HDF cultivated with 5.55 mM, 12 mM or 25 mM glucose or in HDF expressing shGDF15 grown in 5.55 mM glucose. Normalization of the data to GusB (β-glucuronidase), N = 3. **O)** Quantification of PGC1α mRNA transcript by taqman quantitative PCR in HDF transfected with esiNR2F2. Normalization of data to GusB (β-glucuronidase), N = 3. **P,Q)** Quantification of the total Coenzyme Q10 (oxidized and reduced forms) in HDF cultivated with 5.55 mM, 12 mM or 12 mM glucose medium supplemented with 100 nM gdf15. Analysis was also performed in HDF expressing shGDF15 in 5.55 mM glucose. **R)** Quantification of TFAM in HDF cultivated with 5.55 mM glucose medium or medium supplemented with 20pM, 100 pM and 100 nM rGDF15. **S)** Summary of the regulatory network linking TFAM, PGC1α, COQ9 and COQ10. All data are expressed as the mean ± SEM. ∗P < 0.05, ∗∗P < 0.01, ∗∗∗P < 0.001. Ordinary one-way ANOVA with Dunett's test correction was used for panel A, B, F, G, I-M, P–R. Unpaired *t*-test was used for panels E, H and O.

We investigated the role of the observed GDF15 inhibition in high glucose-induced metabolic reprogramming. First, we observed that 100 nM GDF15 supplementation fully reversed the metabolomic alterations induced by 12 mM glucose (Fig. 8A), while the genetic ablation of GDF15 using lenti-shRNA (Fig. S11) reproduced these metabolomic changes (Fig. 8A). The modulatory effect of GDF15 on glucose metabolism was further investigated in human dermal skin fibroblasts by respirometry (Fig. 8B). The results revealed the significant (P value < 0.05) inhibition of mitochondrial respiration in shGDF15 cells and increased respiration following 100 nM GDF15 supplementation (Fig. 8B), supporting the view that this factor positively regulates OXPHOS. A dose-dependent activation of mitochondrial respiration by lower doses of GDF15 was observed between 0.02 nM and 1 nM in cells grown in 5 mM glucose medium, supporting a possible regulatory role in skin physiology and bioenergetics (Fig. 8C). A plateau was reached after 1 nM suggesting that bioenergetic tuning by GDF15 occurs below 1 nM exposure. GDF15 escalating doses addition also triggered an increase in ECAR (Fig. S2C), which might be explained by a higher lactate production or CO_2_ generation by TCA cycle [15]. To determine the impact of GDF15 on chromatin accessibility and potential gene activation, we performed an ATAC-seq analysis. Further analysis of the loci activated by GDF15 (GDF15’ON’) revealed increased chromatin accessibility for a series of oxidative phosphorylation genes (Fig. 8D): NDUFA1, NDUFA4, NDUFA4L2, NDUFB3, NDUFS5P5, COX20, COX4I1P1, COX6C, COX7B, ATP5MC1P1, and ATP5MG. The master regulator of OXPHOS, PPARγ, was also detected in this analysis as well as four mitochondrial ribosomal proteins (MRPL14, MRPL22, MRPL43, MRPS6). Accordingly, Taqman RT‒qPCR experiments showed increased NR2F2, GDF15 and TFAM expression in cells supplemented with 100 nM of GDF15 (Fig. 8E–H). Likewise, human dermal skin fibroblasts overexpressing GDF15 also exhibited increased NDUFB8 (complex I), UQCRC2 (complex III), and COX4 (complex IV) expression and decreased SDHB (complex II) and ATP5A (complex V) expression (Fig. S12), as determined by WES. No effect of GDF15 addition on MTERF3 expression was observed (Fig. 8G).

### 3.10 GDF15 activates PGC1α and TFAM

By using RTqPCR (Taqman), GDF15 supplementation at physiological doses (20–100 pM) activated the expression of selected targets (Fig. 8I–L): GDF15 (self-activation), MAPK1, MAPK3 and PGC1α. A bell-shaped curve was observed for these activations, with a peak at 100 pM. The mechanisms underlying OXPHOS modulation by GDF15 could involve the PGC1α-dependent activation of TFAM [18]. Accordingly, the TFAM mRNA content was reduced in cells exposed to 12 and 25 mM glucose, as was also observed following GDF15 knockdown (Fig. 8M). A proteomic study of cells exposed to 12 mM glucose stress also revealed a reduction in the TFAM protein content (Fig. 2B). PGC1α, the master regulator of mitochondrial biogenesis (mostly through TFAM stimulation), was significantly reduced in cells exposed to 12 and 25 mM glucose, as also observed following GDF15 knockdown (Fig. 8N). Mechanistically, siRNAs targeting NR2F2 induced a significant decrease in PGC1α expression (Fig. 8O), as was also observed for MYCN after siRNAs knockdown (Fig. S7), suggesting the positive regulation of OXPHOS by these two transcription factors. Moreover, TFAM and PGC1α are essential for mitochondrial biogenesis and function, thereby influencing the synthesis of COQ10, which is crucial for the electron transport chain. We observed that shGDF15 HDFs had significantly reduced levels of both oxidized COQ10 and reduced COQ10 and that these effects were reversed by supplementation with purified recombinant GDF15 (Fig. 8P and Q). Likewise, TFAM expression was stimulated by GDF15 used at 20 pM and 100 pM (Fig. 8R). Accordingly, the proteomic analysis of HDFs exposed to high glucose stress showed a reduced expression of COQ9 (Fig. 2A,B), a protein essential for COQ10 biosynthesis. A decrease in PGC1α and TFAM may therefore reduce COQ10 levels, affecting the mitochondrial capacity to produce energy (Fig. 8S). We then evaluated the possible occurrence of mitochondrial damage-associated senescence (MIDAS) in cells exposed to high glucose or in cells expressing a shRNA targeting GDF15. The quantification of different senescence markers such as SA-β-gal activity or p16INK4a and p21 expression did not show significant changes in HDFs grown in high glucose (Fig. S13), at low passages (passages 1–8), in agreement with previous work [16]. Taken together, these results indicate that GDF15 is a positive regulator of OXPHOS in the human dermis and that high-glucose stress contributes to mitochondrial repression through NR2F2-dependent GDF15 expression downregulation and PGC1α-TFAM axis inhibition. At last, we performed a hierarchical clustering analysis to compare the impact of MTERF3 overexpression and GDF15 downregulation on the proteome of HDFs (Fig. S14). A series of clusters showed the difference between these two conditions and revealed groups of proteins specifically upregulated in response to MTERF3 overexpression, and downregulated in response to GDF15 downregulation (Table S7). The changes in MTERF3 and GDF15 expression induced by 12 mM glucose stress were verified on two other human fibroblasts cell lines obtained from independent donors. In these conditions (Fig. S15) we observed a significant decrease in GDF15 and an increase in MTERF3, as initially found on the first donor.

## 4 Discussion

Our study reveals a novel NR2F2–MTERF3–GDF15 axis that represses oxidative phosphorylation (OXPHOS) under high-glucose stress. Exposure to glucose at 6.5 mM (upper normoglycemia boundary) and 12 mM (validated hyperglycemia) modulated GDF15 and MTERF3 expression, highlighting a highly sensitive mitochondrial repression mechanism. While additional transcription factors (e.g., ATF3/4, CHOP, p53) can regulate GDF15 [32], their precise roles under high-glucose conditions warrant further exploration. In our human skin fibroblast (HDF) model, glucose elevation decreased GDF15 expression, in contrast to clinical data showing elevated GDF15 in insulin-resistant or diabetic patients. This discrepancy may reflect the inflammatory processes in diabetes that drive GDF15 upregulation [37], as GDF15 also rises in preeclampsia, depression, heart failure, and kidney injury [33,[38], [39], [40], [41], [42]]. Tissue specificity may also play a role, since liver and kidney produce more GDF15 in diabetic patients, and our *in vitro* exposure was only 48 h, not fully replicating chronic hyperglycemia. Additionally, peripheral GDF15 resistance could occur in diabetic states, similar to insulin resistance. We found that 12 mM glucose induces: (i) A GDF15-dependent reduction in PGC1α–TFAM-driven mitochondrial biogenesis and (ii) An MTERF3-dependent decline in OXPHOS protein content. Furthermore, NR2F2 (COUP-TFII) emerged as an upstream regulator of both GDF15 and MTERF3, suggesting a coordinated mechanism of OXPHOS repression in high-glucose conditions. Previous studies associate GDF15 with compensatory OXPHOS biogenesis following respiratory chain inhibition, partly via AMPK–PGC1α pathways [14,17,[19], [20], [21],[43], [44], [45]]. Consistently, we observed that physiologically relevant GDF15 doses (0.02–1 nM, i.e., 0.6–30 ng/mL) stimulated mitochondrial biogenesis and respiration in human dermal fibroblasts, increasing mitochondrial tubule length and branching. This finding aligns with potential therapeutic benefits of GDF15 in cardiac hypertrophy and hepatic steatosis [22,46]. Notably, different GDF15 doses were used here: a higher, supraphysiological concentration (100 nM or 3000 ng/mL) effectively rescued hyperglycemia-induced metabolomic changes, while low nanomolar doses relevant to normal or mildly elevated plasma levels enhanced OXPHOS function. Elevated GDF15 (4 ng/mL range) in mitochondrial disorders may partially mitigate dysfunction in tissues, complementing GDF15's known central (brain) effects [47]. GDF15 analogs are under clinical evaluation for cardiometabolic diseases and obesity [32,48], whereas monoclonal antibodies have been tested in cancer-associated cachexia [49]. The impact of such interventions on mitochondrial function remains to be fully explored. In our work, a simple WES technique (differing from Western blot) helped detect GDF15 in tissues but produced apparent molecular weights that differ from traditional WB data. Additionally, we found that high glucose inhibited NR2F2, which in turn suppressed GDF15 and many subunits of respiratory complexes I–V. FOS also negatively regulated GDF15, though the potential cooperative effect of NR2F2 and FOS on OXPHOS requires further study. NR2F2 may even modulate other GDF15 regulators (ATF3, ATF4, CHOP, p53), indicating a multifactorial control of GDF15 in hyperglycemia. Using a range of high-throughput techniques, we observed that GDF15 downregulation drastically changed chromatin accessibility in ∼4000 genomic loci, consistent with broad regulatory effects. This differs from a prior report (912 genes altered) [50], potentially due to ATAC-seq's unique detection of chromatin accessibility versus RNA-seq's direct measurement of mRNA levels.

Our findings clarify the role of MTERF3 as a Repressor of Mitochondrial Physiology. Biochemical evidence indicates MTERF3 modulates mitochondrial DNA transcription, ribosomal assembly, and is part of RNA granule complexes [23,24,26,51,52]. In our system, high glucose only modestly increased MTERF3 compared to stronger overexpression via a CMV-driven plasmid. Consequently, MTERF3 overexpression inhibited mitochondrial proteins involved in translation (mitoribosomes), cristae organization (MICOS), dynamics, and respiratory complexes. It also increased Jun, a transcription factor involved in cell proliferation, apoptosis, and morphogenesis [53]. However, the precise mechanism linking MTERF3 and Jun remains unclear. Mechanistically, MTERF3 overexpression alone recapitulated the OXPHOS inhibition but did not mimic the full hyperglycemia-induced metabolomic shifts. In contrast, GDF15 inhibition reproduced those metabolomic changes, while exogenous GDF15 rescued them. Also, variations occurred among different dermal systems (HDFs, HED, HRS) regarding OXPHOS protein alterations under high glucose, possibly reflecting keratinocyte involvement or more complex cell–matrix interactions.

We propose that combined MTERF3–GDF15 modulation under 12 mM glucose is a robust way to suppress oxidative phosphorylation. At higher glucose (25 mM), oxidative stress and mitophagy become prominent, suggesting a stepwise intensification of mitochondrial repression and degradation with rising glucose. These findings have practical implications for cell culture protocols using 25 mM glucose, which may bias mitochondrial or redox studies. We observed no induction of senescence under our conditions, consistent with Wedel et al. [16], who showed that GDF15 knockdown increased senescence only after multiple passages. Our work identifies NR2F2 as a dual controller of GDF15 and MTERF3. Genetic downregulation of NRF2 by esiRNA led to MTERF3 overexpression, as shown by TaqMan RT-qPCR and a MTERF3 promoter activation assay. Likewise, reducing NR2F2 levels via esiRNA or CRISPR-Cas9 knockout lowered GDF15 expression, as measured by TaqMan RT-qPCR. Based on these observations, we propose that NR2F2 acts as a dual regulator of both GDF15 and MTERF3. The functional impact on mitochondrial respiration was demonstrated in three ways: (1) CRISPR-Cas9–mediated NR2F2 downregulation inhibited respiration, (2) MTERF3 overexpression produced the same effect, and (3) shRNA-mediated GDF15 knockdown (shGDF15) similarly impaired respiration. Conversely, supplementing with GDF15 activated mitochondrial respiration. The MTERF3 promoter contains an NR2F2-binding site, and *in silico* analyses show NR2F2 targets (MTERF1–4 and GDF1–2,5,9,11,15; ENCODE dataset). At last, NR2F2 expression decreases under high glucose in mouse pancreas and liver [30,54], corroborating our findings in human skin fibroblasts.

High-glucose stress also affected dermal and epidermal structure. Fibroblast haptotaxis declined under hyperglycemia or when GDF15 or TFAM were silenced, and skin reconstruction was blocked by GDF15 knockdown. The collagen network produced by fibroblasts was similarly compromised by hyperglycemia or GDF15 downregulation. Thus, our data reveal a concerted repression of mitochondrial function in the human dermis driven by the interplay among NR2F2, MTERF3, and GDF15, offering a new perspective on hyperglycemia-induced tissue alterations. To conclude, this study defines a coordinated regulatory mechanism wherein high glucose suppresses NR2F2, reducing GDF15 and upregulating MTERF3, collectively inhibiting the mitochondrial proteome and function in dermal fibroblasts. While GDF15 may rescue aspects of metabolic dysfunction, chronic hyperglycemia *in vivo* involves additional inflammatory pathways that may override this protective response. Understanding the interplay of NR2F2, MTERF3, and GDF15 broadens our grasp of hyperglycemia's tissue-specific impacts and may inform future therapeutic strategies targeting mitochondrial health.

## CRediT authorship contribution statement

**S. Ley-Ngardigal:** Writing – review & editing, Writing – original draft, Project administration, Methodology, Investigation, Formal analysis, Data curation, Conceptualization. **S. Claverol:** Validation, Investigation, Formal analysis. **L. Sobilo:** Validation, Methodology, Investigation. **M. Moreau:** Methodology, Investigation. **C. Hubert:** Methodology, Investigation. **J. Goupil:** Methodology, Investigation. **A. Poulignon:** Methodology, Investigation. **W. Mahfouf:** Methodology, Investigation. **H. Fatrouni:** Methodology, Investigation. **L. Dard:** Methodology, Investigation. **M. Juan:** Methodology. **L. Gales:** Methodology, Investigation. **A. Merched:** Resources. **C. Tokarski:** Methodology. **E. Leblanc:** Methodology. **A. Galinier:** Methodology, Investigation. **D. Lacombe:** Resources. **H.R. Rezvani:** Methodology. **F. Bellvert:** Methodology, Investigation. **K. Pays:** Resources. **C. Nizard:** Resources. **N. Dias Amoedo:** Writing – review & editing, Writing – original draft, Validation, Project administration, Methodology, Investigation, Data curation, Conceptualization. **A.L. Bulteau:** Writing – review & editing, Resources. **R. Rossignol:** Writing – review & editing, Writing – original draft, Validation, Supervision, Resources, Project administration, Funding acquisition, Formal analysis, Data curation, Conceptualization.

## Funding

We acknowledge funding by LVMH recherche, Institut National de la Santé et de la Recherche Médicale (10.13039/501100001677Inserm), the 10.13039/501100006251University of Bordeaux, Fondation pour la Recherche médicale (équipe 10.13039/501100002915FRM 2023; EQU202303016289), 10.13039/501100015760INSERM (équipement pour la cancérologie) and Région Nouvelle Aquitaine.

## Declaration of competing interest

CN, EL, SL and ALB are LVMH recherche employees. SLNG received a PH.D grant from LVMH research to perform research at the 10.13039/501100006251University of Bordeaux-INSERM U1211 (grant agreement AST-2021-395). Other authors declare no conflict of interest.

## Data Availability

Data will be made available on request.
