# Supplementary material for: Repression of oxidative phosphorylation by NR2F2, MTERF3 and GDF15 in human skin under high-glucose stress
Source: Redox Biol. 2025 Mar 27;82:103613. doi: 10.1016/j.redox.2025.103613 (PMC11999475; doi:10.1016/j.redox.2025.103613)
Supplement: Multimedia component 2 [file mmc2.pptx]

## Slide 1
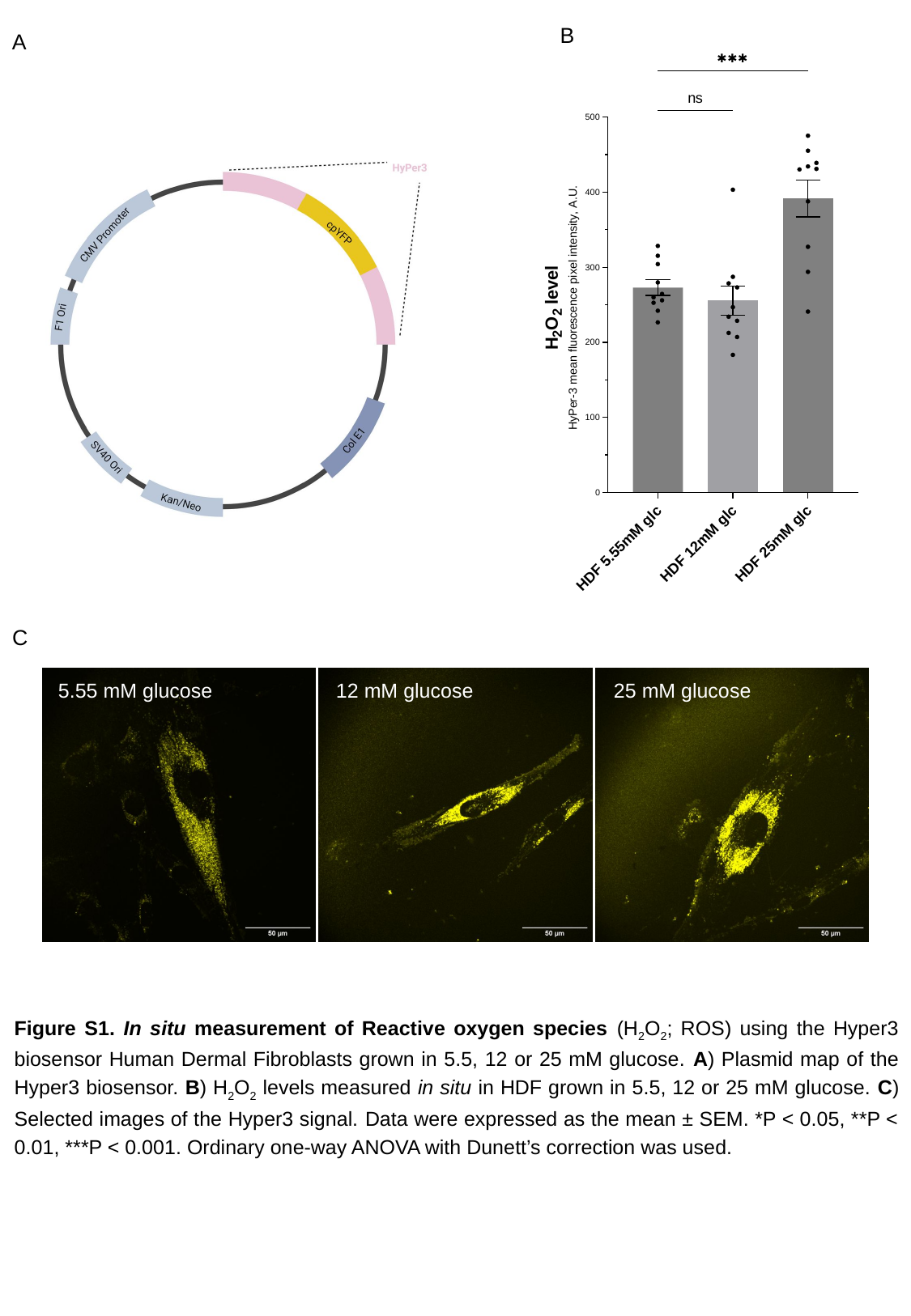

B
A
C
5.55 mM glucose
12 mM glucose
25 mM glucose
Figure S1. In situ measurement of Reactive oxygen species (H2O2; ROS) using the Hyper3 biosensor Human Dermal Fibroblasts grown in 5.5, 12 or 25 mM glucose. A) Plasmid map of the Hyper3 biosensor. B) H2O2 levels measured in situ in HDF grown in 5.5, 12 or 25 mM glucose. C) Selected images of the Hyper3 signal. Data were expressed as the mean ± SEM. *P < 0.05, **P < 0.01, ***P < 0.001. Ordinary one-way ANOVA with Dunett’s correction was used.

## Slide 2
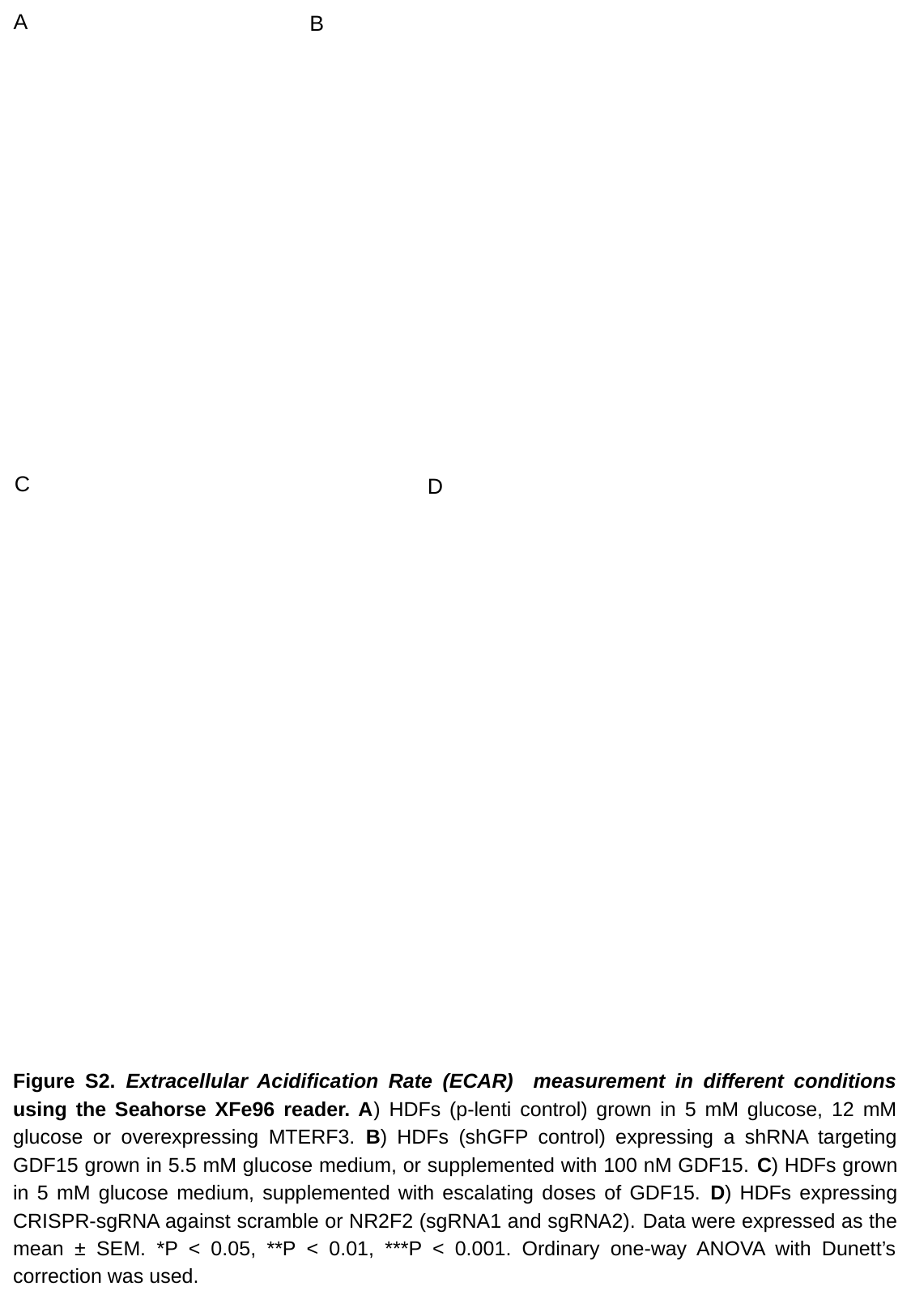

A
B
C
D
Figure S2. Extracellular Acidification Rate (ECAR) measurement in different conditions using the Seahorse XFe96 reader. A) HDFs (p-lenti control) grown in 5 mM glucose, 12 mM glucose or overexpressing MTERF3. B) HDFs (shGFP control) expressing a shRNA targeting GDF15 grown in 5.5 mM glucose medium, or supplemented with 100 nM GDF15. C) HDFs grown in 5 mM glucose medium, supplemented with escalating doses of GDF15. D) HDFs expressing CRISPR-sgRNA against scramble or NR2F2 (sgRNA1 and sgRNA2). Data were expressed as the mean ± SEM. *P < 0.05, **P < 0.01, ***P < 0.001. Ordinary one-way ANOVA with Dunett’s correction was used.

## Slide 3
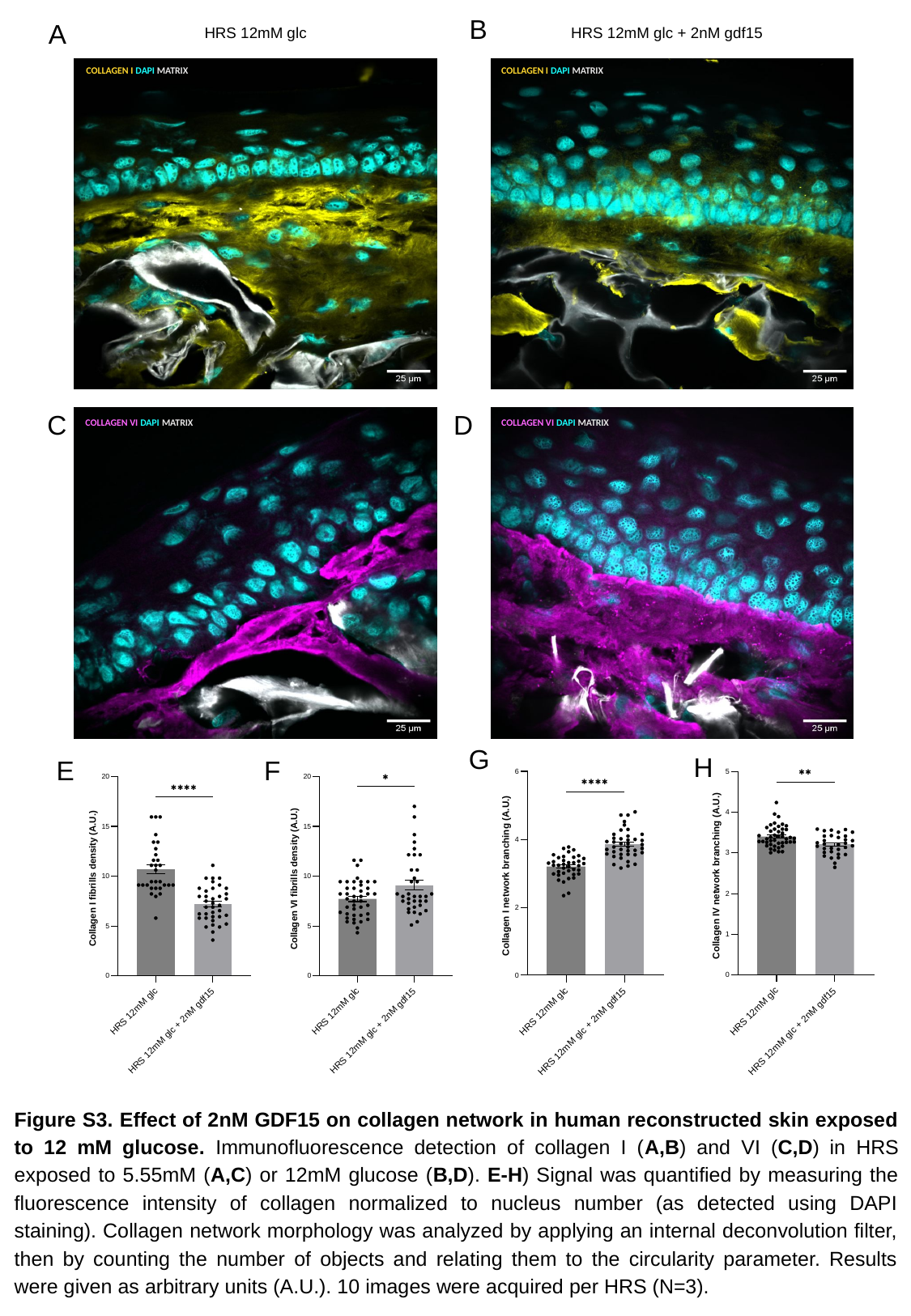

B
A
HRS 12mM glc
HRS 12mM glc + 2nM gdf15
COLLAGEN I DAPI MATRIX
COLLAGEN I DAPI MATRIX
COLLAGEN VI DAPI MATRIX
COLLAGEN VI DAPI MATRIX
D
C
G
H
E
F
Figure S3. Effect of 2nM GDF15 on collagen network in human reconstructed skin exposed to 12 mM glucose. Immunofluorescence detection of collagen I (A,B) and VI (C,D) in HRS exposed to 5.55mM (A,C) or 12mM glucose (B,D). E-H) Signal was quantified by measuring the fluorescence intensity of collagen normalized to nucleus number (as detected using DAPI staining). Collagen network morphology was analyzed by applying an internal deconvolution filter, then by counting the number of objects and relating them to the circularity parameter. Results were given as arbitrary units (A.U.). 10 images were acquired per HRS (N=3).

## Slide 4
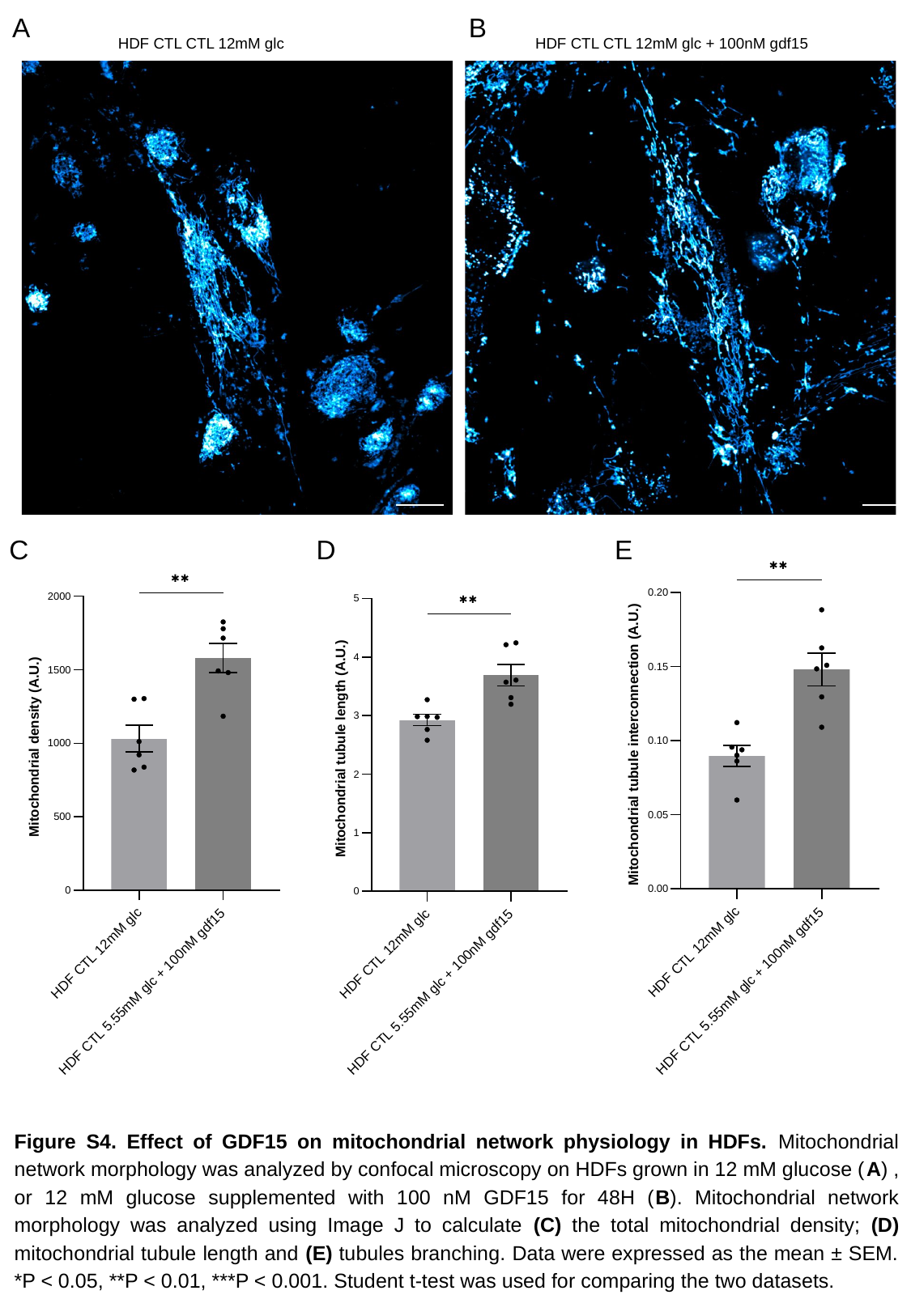

A
B
HDF CTL CTL 12mM glc
HDF CTL CTL 12mM glc + 100nM gdf15
C
D
E
Figure S4. Effect of GDF15 on mitochondrial network physiology in HDFs. Mitochondrial network morphology was analyzed by confocal microscopy on HDFs grown in 12 mM glucose (A) , or 12 mM glucose supplemented with 100 nM GDF15 for 48H (B). Mitochondrial network morphology was analyzed using Image J to calculate (C) the total mitochondrial density; (D) mitochondrial tubule length and (E) tubules branching. Data were expressed as the mean ± SEM. *P < 0.05, **P < 0.01, ***P < 0.001. Student t-test was used for comparing the two datasets.

## Slide 5
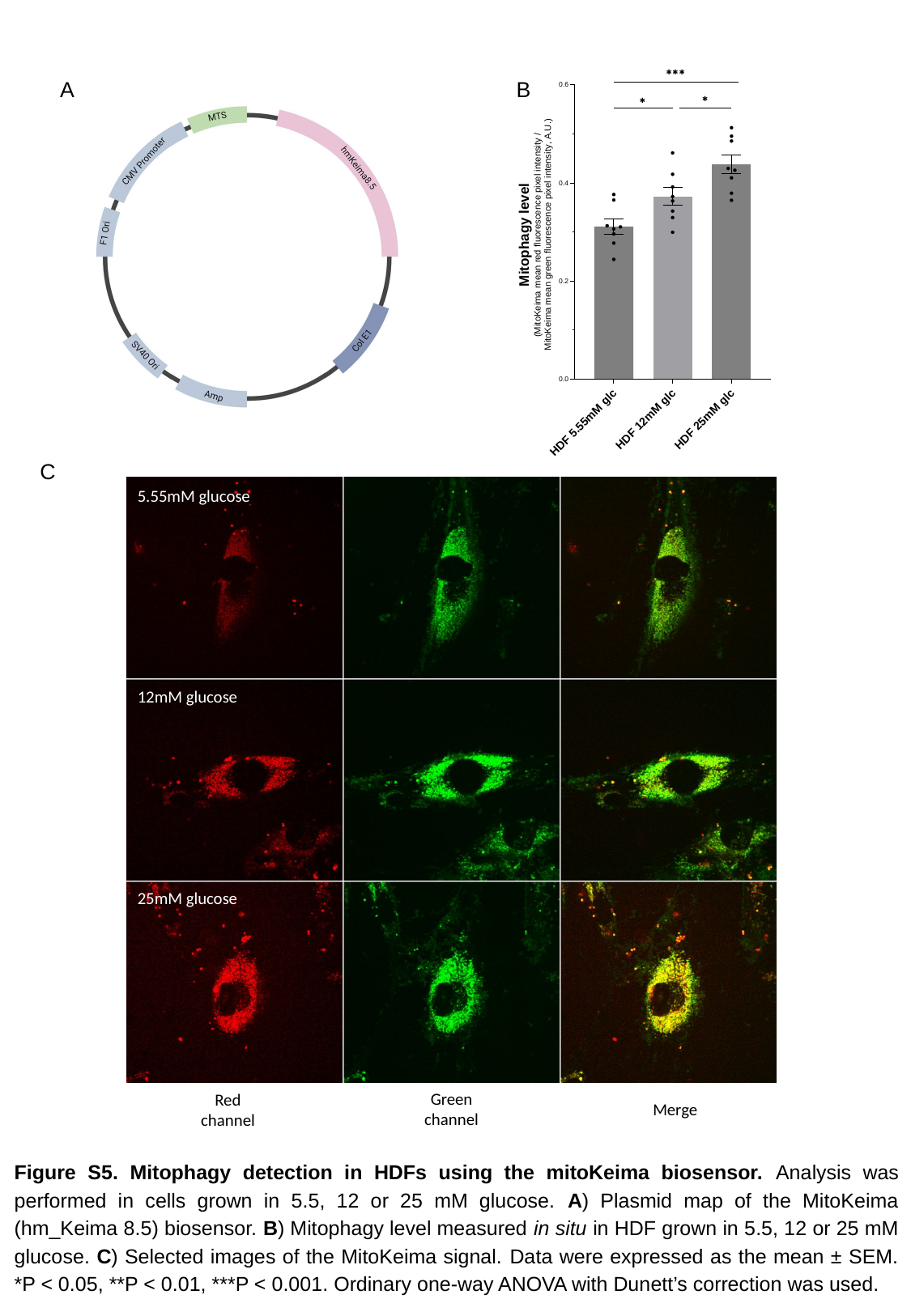

A
B
C
5.55mM glucose
12mM glucose
25mM glucose
Green channel
Red channel
Merge
Figure S5. Mitophagy detection in HDFs using the mitoKeima biosensor. Analysis was performed in cells grown in 5.5, 12 or 25 mM glucose. A) Plasmid map of the MitoKeima (hm_Keima 8.5) biosensor. B) Mitophagy level measured in situ in HDF grown in 5.5, 12 or 25 mM glucose. C) Selected images of the MitoKeima signal. Data were expressed as the mean ± SEM. *P < 0.05, **P < 0.01, ***P < 0.001. Ordinary one-way ANOVA with Dunett’s correction was used.

## Slide 6
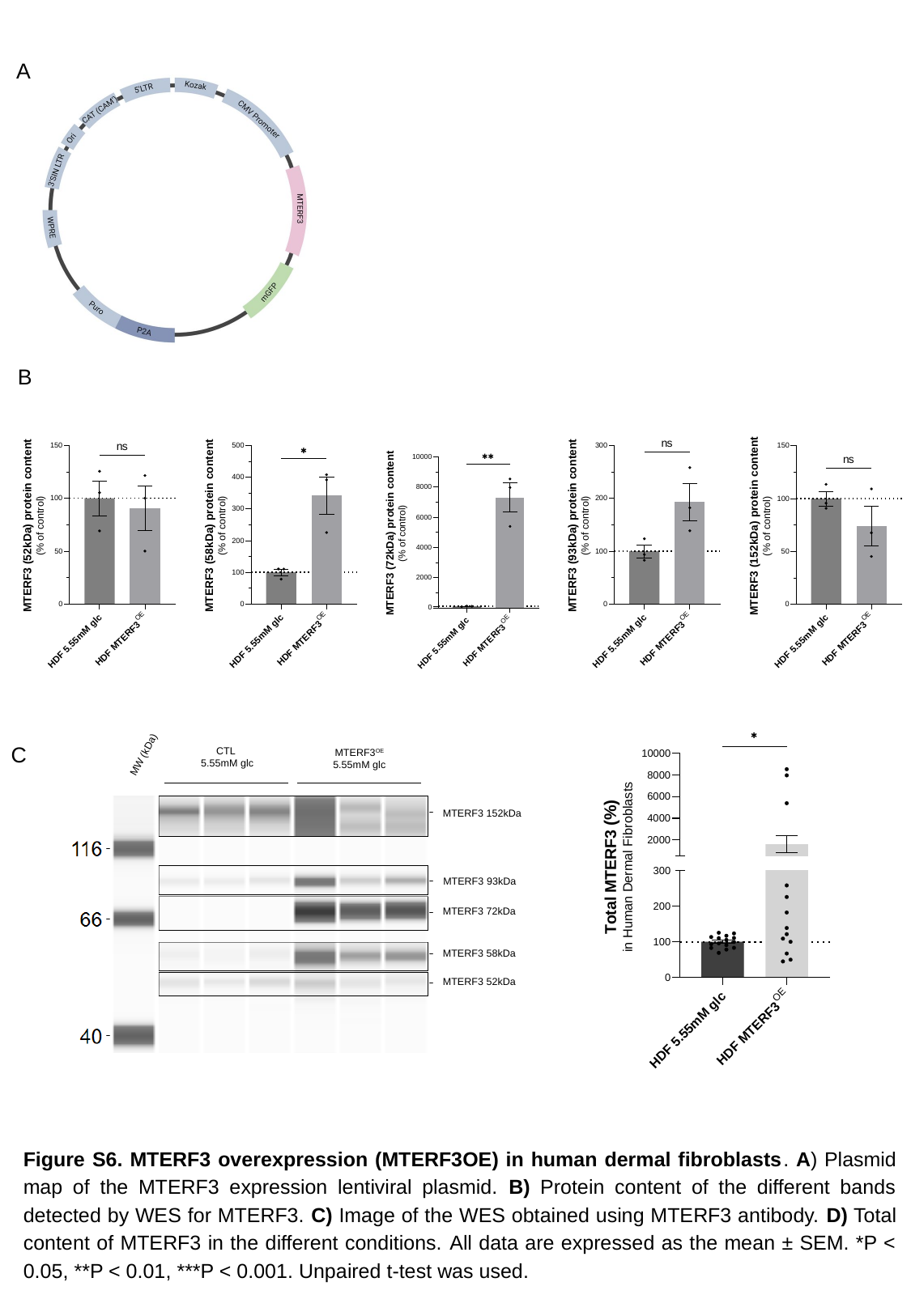

A
B
CTL
5.55mM glc
MTERF3OE
5.55mM glc
MW (kDa)
MTERF3 152kDa
MTERF3 93kDa
MTERF3 72kDa
MTERF3 58kDa
MTERF3 52kDa
C
Figure S6. MTERF3 overexpression (MTERF3OE) in human dermal fibroblasts. A) Plasmid map of the MTERF3 expression lentiviral plasmid. B) Protein content of the different bands detected by WES for MTERF3. C) Image of the WES obtained using MTERF3 antibody. D) Total content of MTERF3 in the different conditions. All data are expressed as the mean ± SEM. *P < 0.05, **P < 0.01, ***P < 0.001. Unpaired t-test was used.

## Slide 7
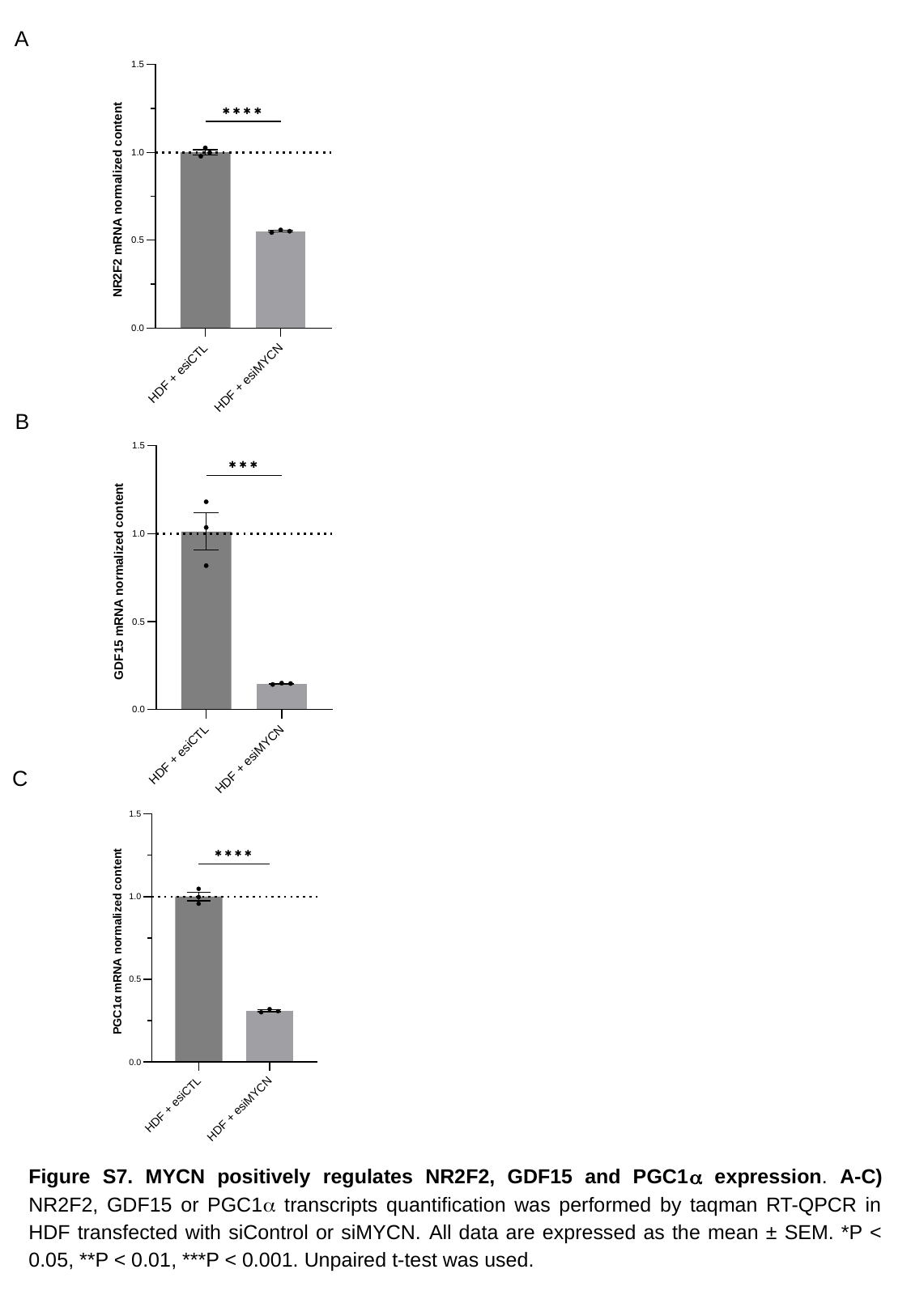

A
B
C
Figure S7. MYCN positively regulates NR2F2, GDF15 and PGC1a expression. A-C) NR2F2, GDF15 or PGC1a transcripts quantification was performed by taqman RT-QPCR in HDF transfected with siControl or siMYCN. All data are expressed as the mean ± SEM. *P < 0.05, **P < 0.01, ***P < 0.001. Unpaired t-test was used.

## Slide 8
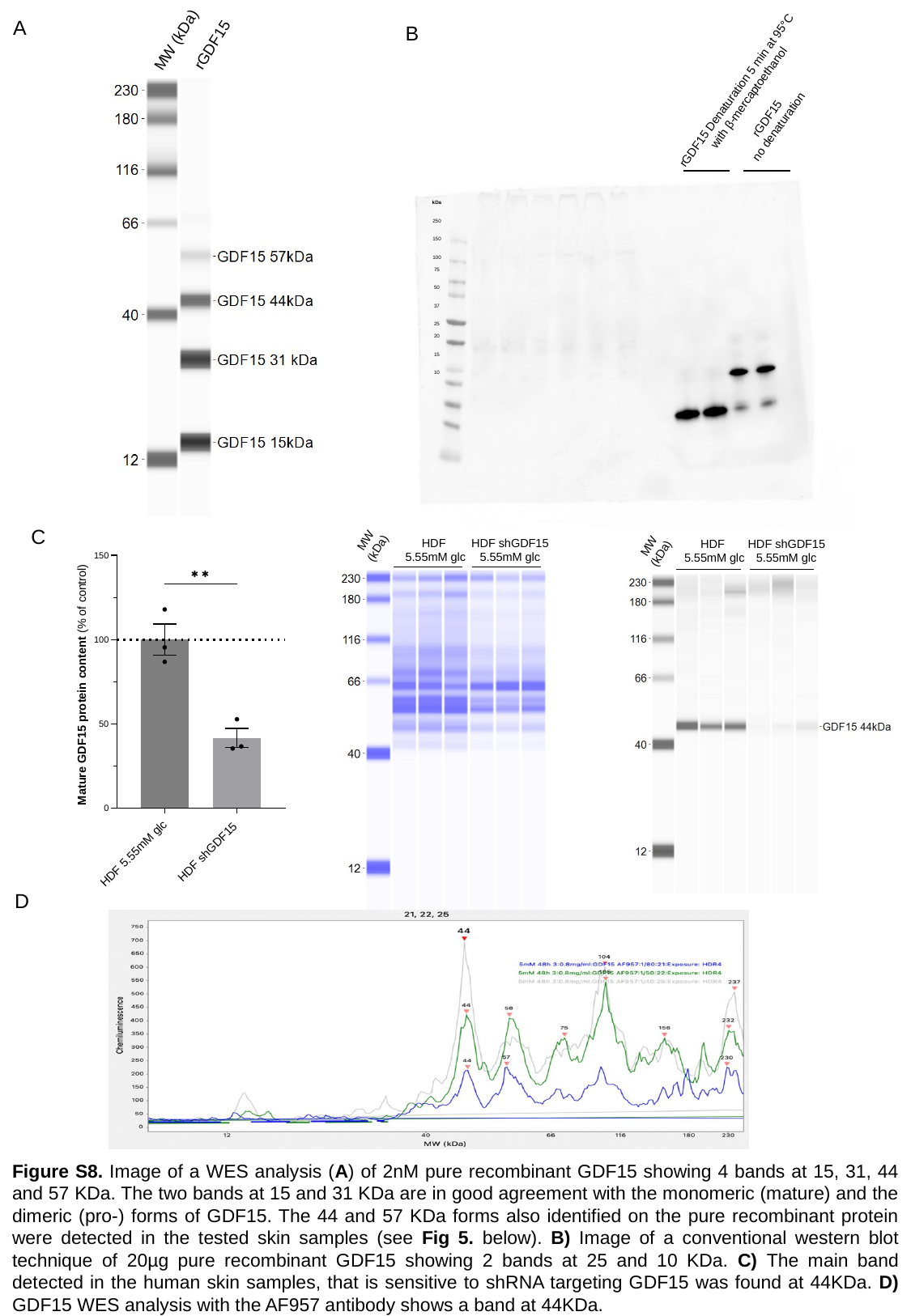

rGDF15 Denaturation 5 min at 95°C
with β-mercaptoethanol
rGDF15
no denaturation
kDa
250
150
100
75
50
37
25
20
15
10
MW (kDa)
A
B
rGDF15
HDF shGDF15
5.55mM glc
MW (kDa)
HDF
5.55mM glc
C
MW (kDa)
HDF
5.55mM glc
HDF shGDF15
5.55mM glc
D
Figure S8. Image of a WES analysis (A) of 2nM pure recombinant GDF15 showing 4 bands at 15, 31, 44 and 57 KDa. The two bands at 15 and 31 KDa are in good agreement with the monomeric (mature) and the dimeric (pro-) forms of GDF15. The 44 and 57 KDa forms also identified on the pure recombinant protein were detected in the tested skin samples (see Fig 5. below). B) Image of a conventional western blot technique of 20µg pure recombinant GDF15 showing 2 bands at 25 and 10 KDa. C) The main band detected in the human skin samples, that is sensitive to shRNA targeting GDF15 was found at 44KDa. D) GDF15 WES analysis with the AF957 antibody shows a band at 44KDa.

## Slide 9
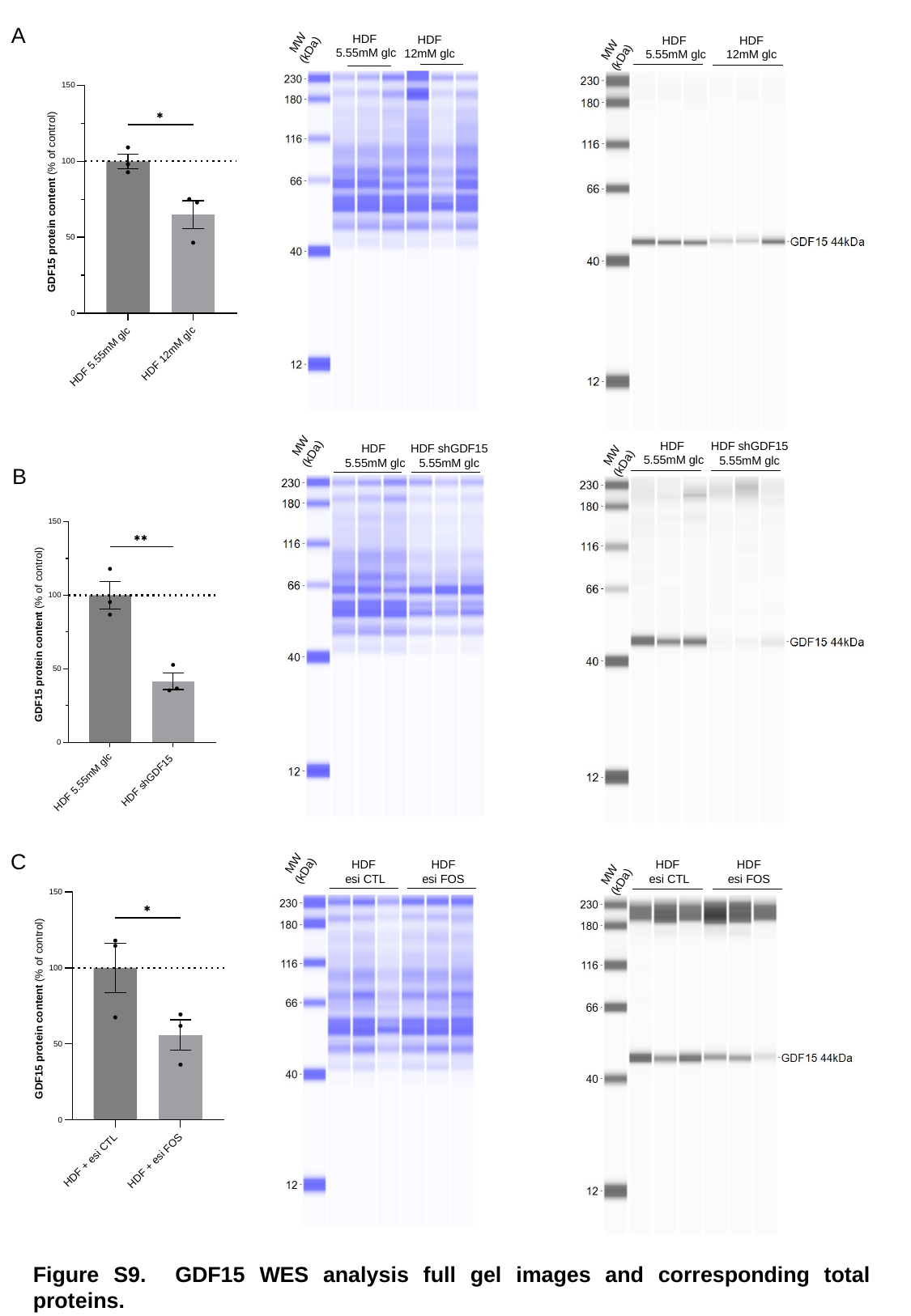

A
HDF
5.55mM glc
HDF
12mM glc
MW (kDa)
HDF
5.55mM glc
MW (kDa)
HDF
12mM glc
HDF shGDF15
5.55mM glc
HDF
5.55mM glc
MW (kDa)
HDF shGDF15
5.55mM glc
MW (kDa)
HDF
5.55mM glc
B
C
HDF
esi CTL
HDF
esi FOS
MW (kDa)
MW (kDa)
HDF
esi CTL
HDF
esi FOS
Figure S9. GDF15 WES analysis full gel images and corresponding total proteins.

## Slide 10
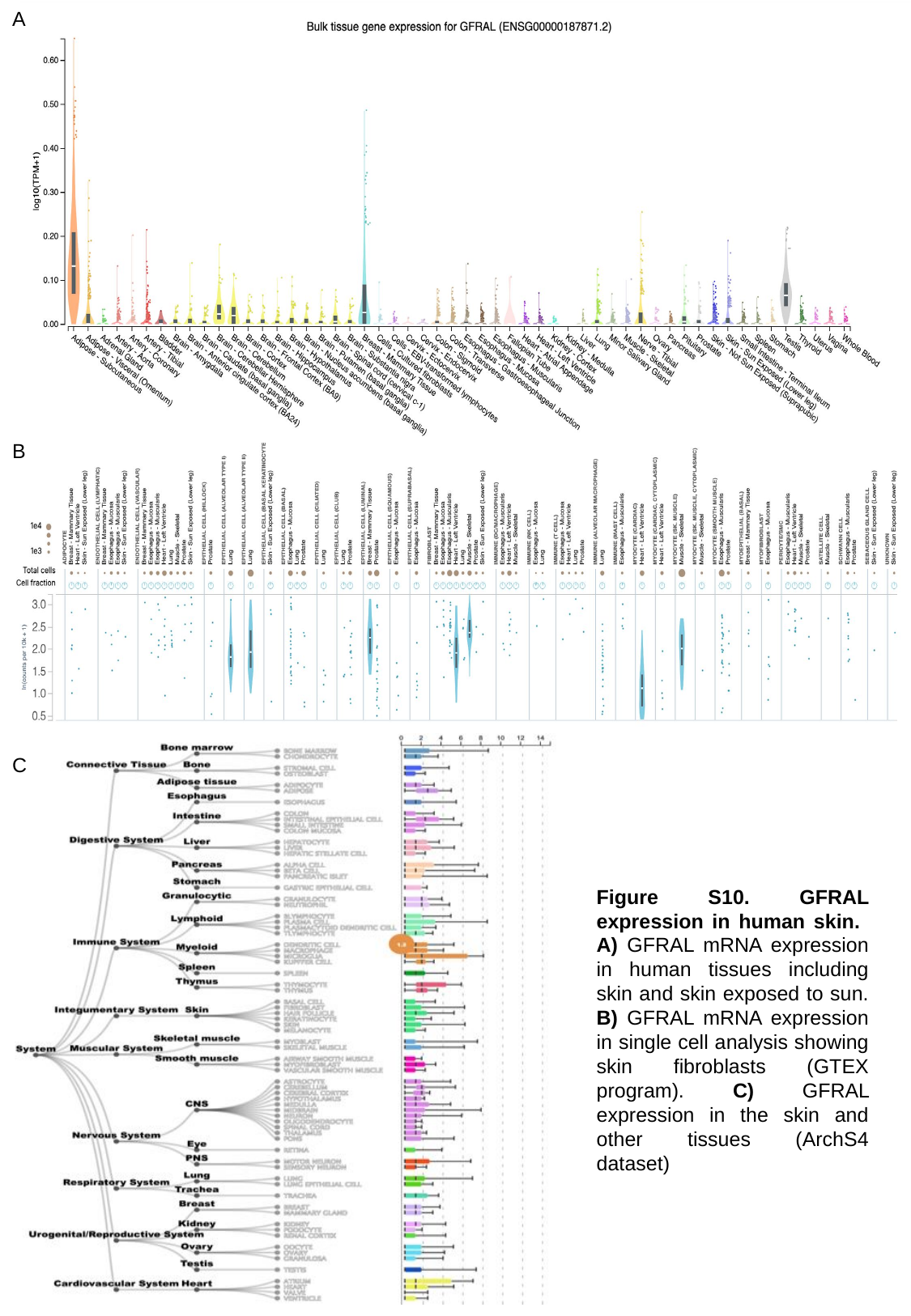

A
B
C
Figure S10. GFRAL expression in human skin. A) GFRAL mRNA expression in human tissues including skin and skin exposed to sun. B) GFRAL mRNA expression in single cell analysis showing skin fibroblasts (GTEX program). C) GFRAL expression in the skin and other tissues (ArchS4 dataset)

## Slide 11
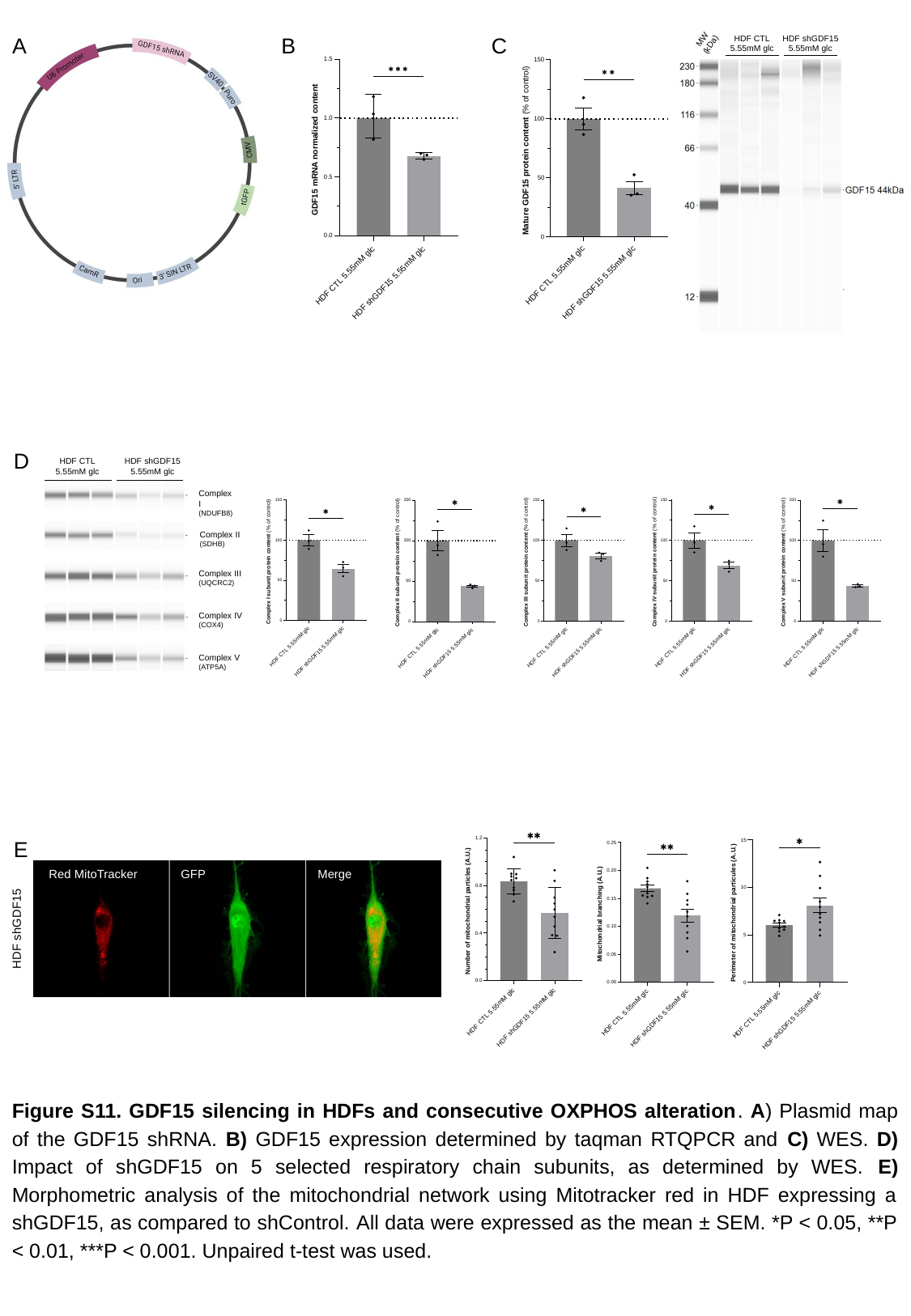

MW (kDa)
HDF CTL
5.55mM glc
HDF shGDF15
5.55mM glc
A
B
C
D
HDF CTL
5.55mM glc
HDF shGDF15
5.55mM glc
Complex I (NDUFB8)
Complex II
(SDHB)
Complex III
(UQCRC2)
Complex IV
(COX4)
Complex V
(ATP5A)
E
Merge
GFP
Red MitoTracker
HDF shGDF15
Figure S11. GDF15 silencing in HDFs and consecutive OXPHOS alteration. A) Plasmid map of the GDF15 shRNA. B) GDF15 expression determined by taqman RTQPCR and C) WES. D) Impact of shGDF15 on 5 selected respiratory chain subunits, as determined by WES. E) Morphometric analysis of the mitochondrial network using Mitotracker red in HDF expressing a shGDF15, as compared to shControl. All data were expressed as the mean ± SEM. *P < 0.05, **P < 0.01, ***P < 0.001. Unpaired t-test was used.

## Slide 12
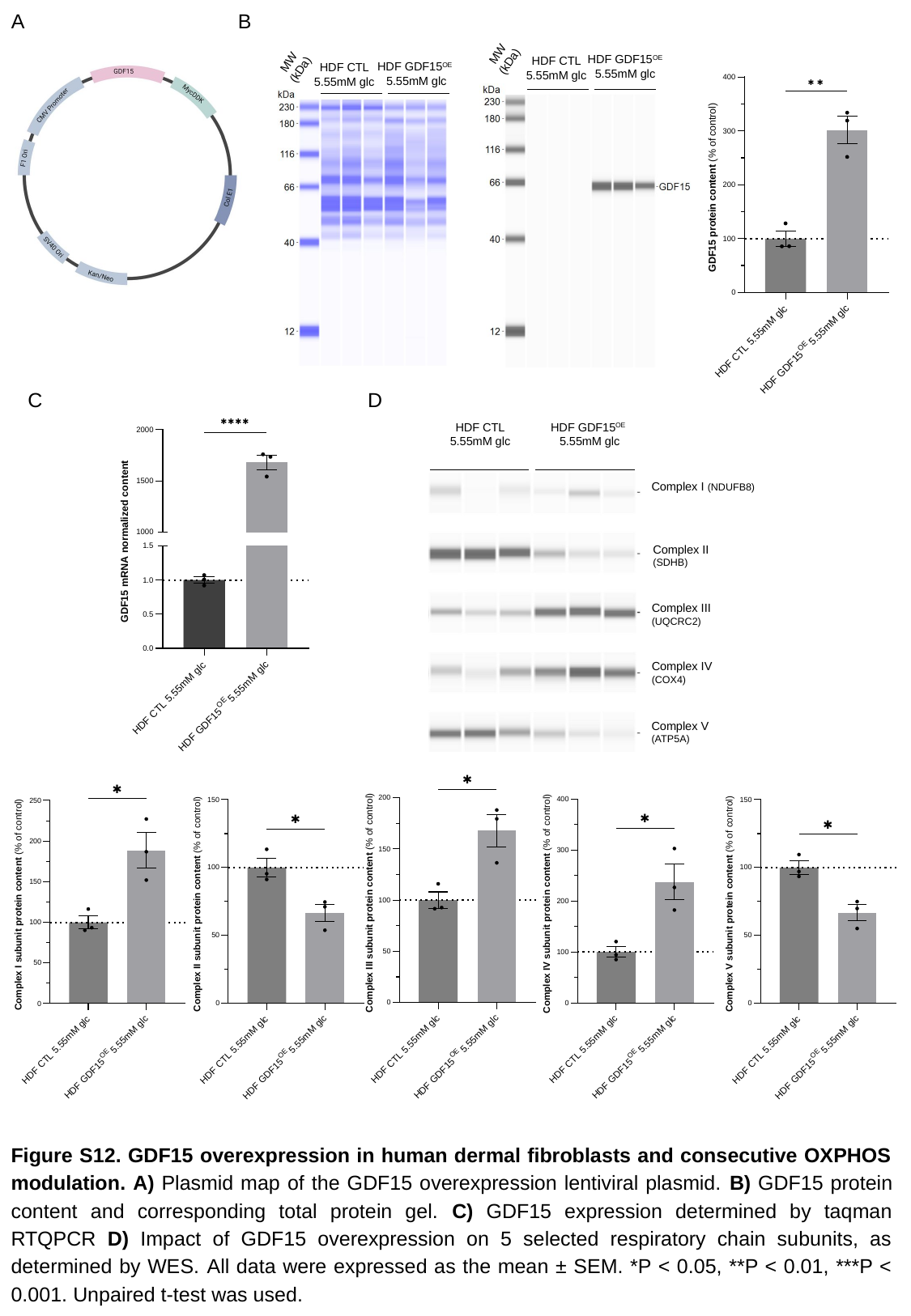

A
B
MW (kDa)
HDF GDF15OE
5.55mM glc
HDF CTL
5.55mM glc
MW (kDa)
HDF GDF15OE
5.55mM glc
HDF CTL
5.55mM glc
D
C
HDF CTL
5.55mM glc
HDF GDF15OE
5.55mM glc
Complex I (NDUFB8)
Complex II
(SDHB)
Complex III
(UQCRC2)
Complex IV
(COX4)
Complex V
(ATP5A)
Figure S12. GDF15 overexpression in human dermal fibroblasts and consecutive OXPHOS modulation. A) Plasmid map of the GDF15 overexpression lentiviral plasmid. B) GDF15 protein content and corresponding total protein gel. C) GDF15 expression determined by taqman RTQPCR D) Impact of GDF15 overexpression on 5 selected respiratory chain subunits, as determined by WES. All data were expressed as the mean ± SEM. *P < 0.05, **P < 0.01, ***P < 0.001. Unpaired t-test was used.

## Slide 13
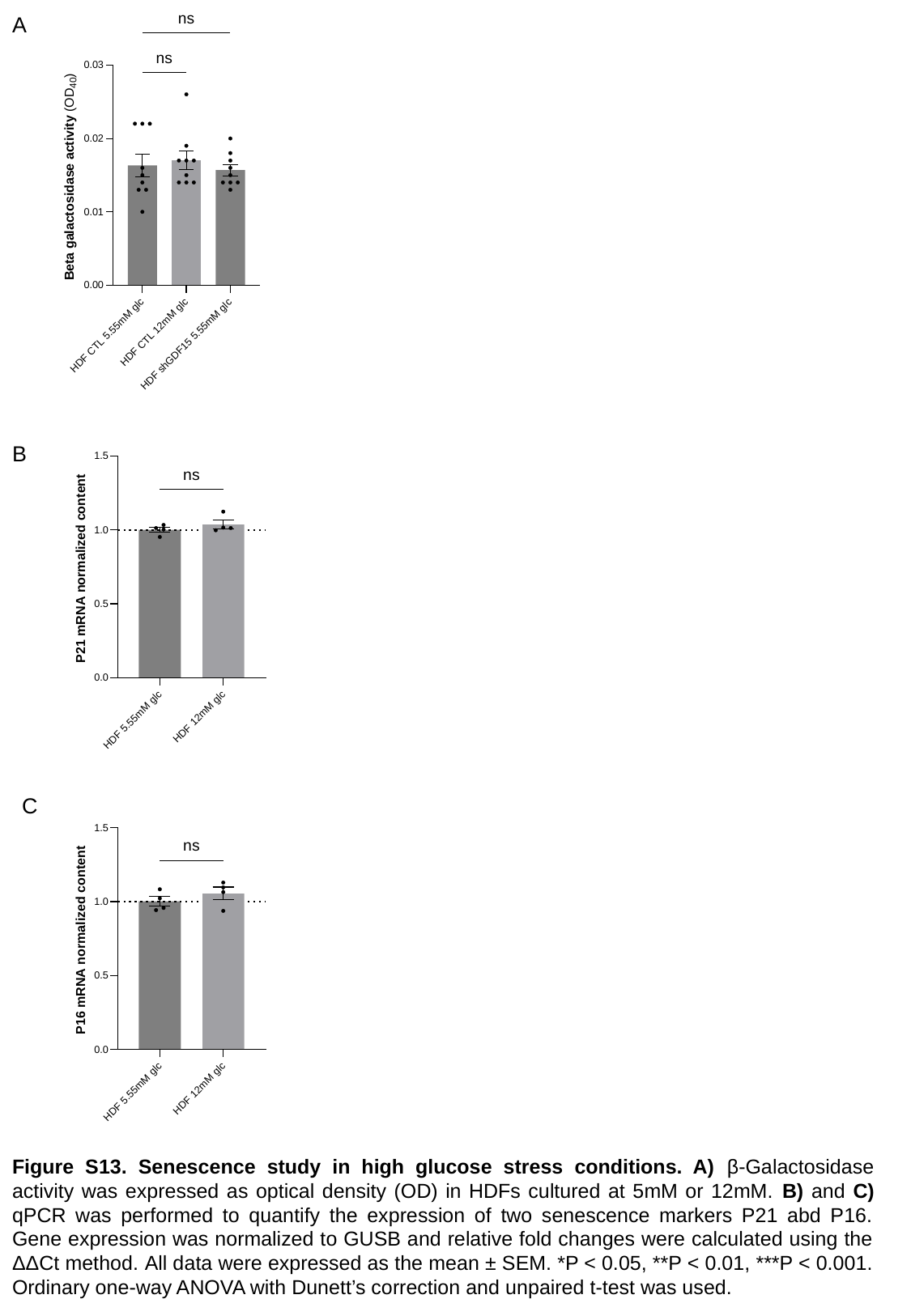

A
B
C
Figure S13. Senescence study in high glucose stress conditions. A) β-Galactosidase activity was expressed as optical density (OD) in HDFs cultured at 5mM or 12mM. B) and C) qPCR was performed to quantify the expression of two senescence markers P21 abd P16. Gene expression was normalized to GUSB and relative fold changes were calculated using the ΔΔCt method. All data were expressed as the mean ± SEM. *P < 0.05, **P < 0.01, ***P < 0.001. Ordinary one-way ANOVA with Dunett’s correction and unpaired t-test was used.

## Slide 14
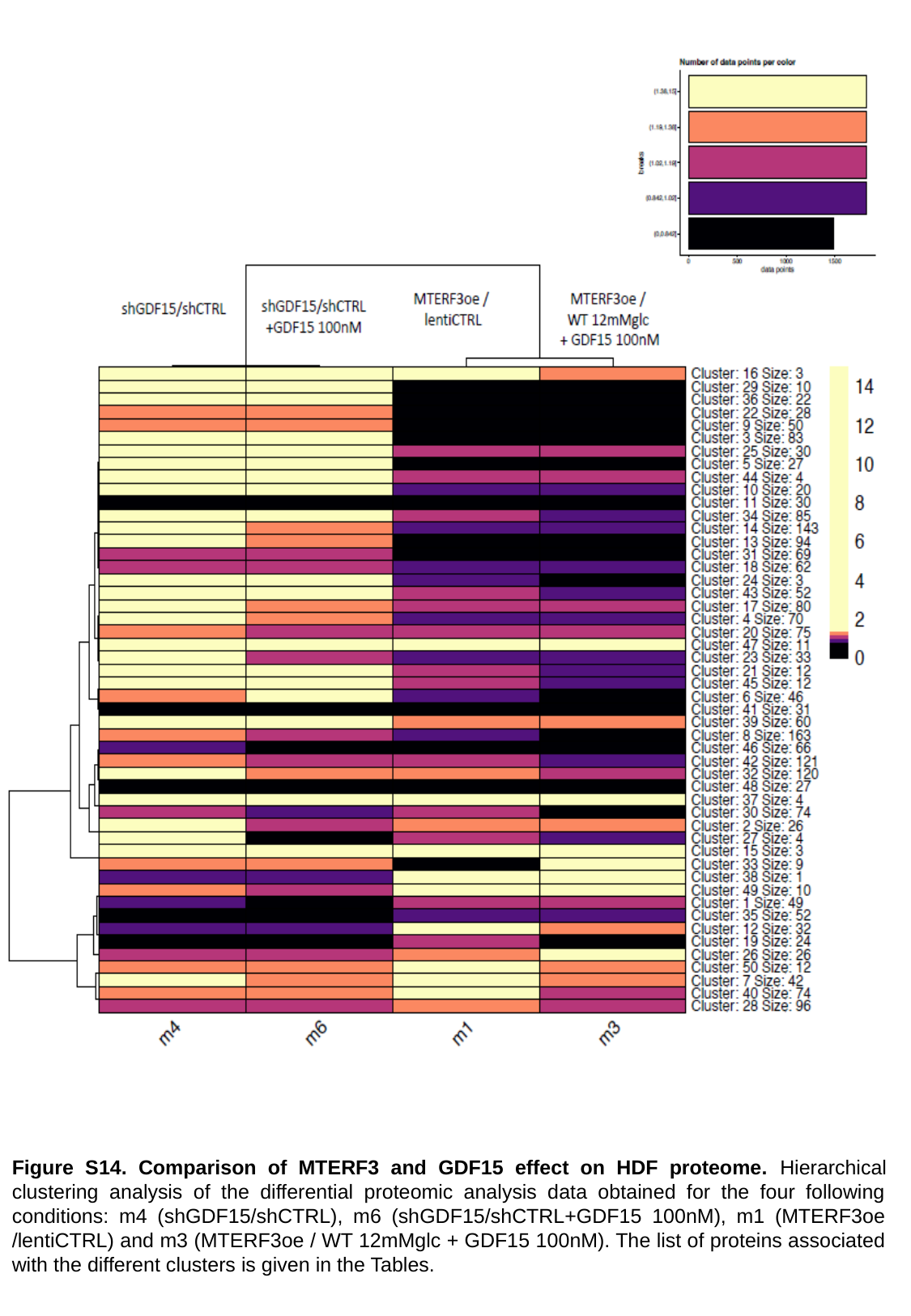

Figure S14. Comparison of MTERF3 and GDF15 effect on HDF proteome. Hierarchical clustering analysis of the differential proteomic analysis data obtained for the four following conditions: m4 (shGDF15/shCTRL), m6 (shGDF15/shCTRL+GDF15 100nM), m1 (MTERF3oe /lentiCTRL) and m3 (MTERF3oe / WT 12mMglc + GDF15 100nM). The list of proteins associated with the different clusters is given in the Tables.

## Slide 15
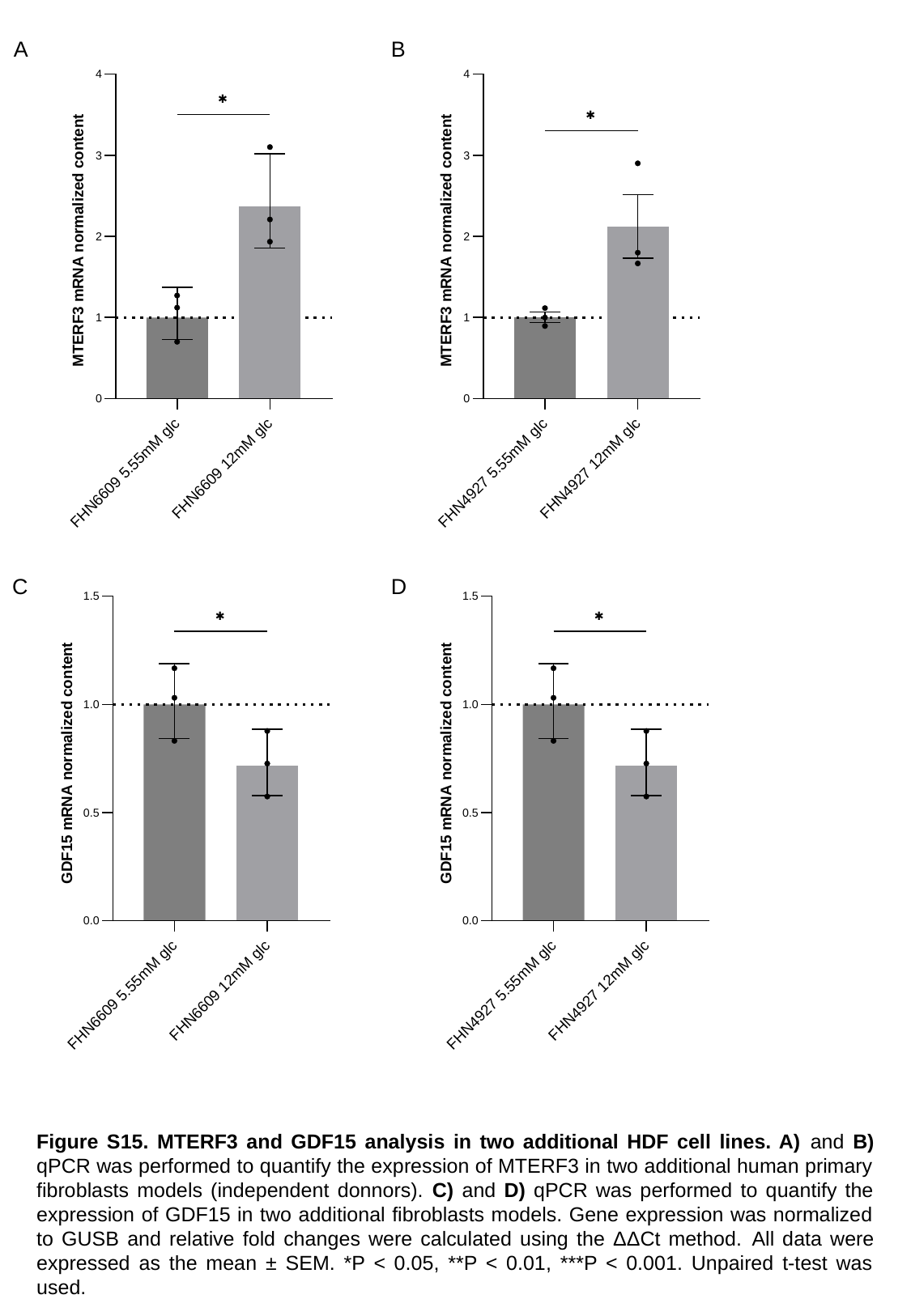

A
B
D
C
Figure S15. MTERF3 and GDF15 analysis in two additional HDF cell lines. A) and B) qPCR was performed to quantify the expression of MTERF3 in two additional human primary fibroblasts models (independent donnors). C) and D) qPCR was performed to quantify the expression of GDF15 in two additional fibroblasts models. Gene expression was normalized to GUSB and relative fold changes were calculated using the ΔΔCt method. All data were expressed as the mean ± SEM. *P < 0.05, **P < 0.01, ***P < 0.001. Unpaired t-test was used.
